# Phenothiazine dye-loaded chitosan cryogels as multifunctional antibacterial wound dressings

**DOI:** 10.1038/s41598-026-55319-w

**Published:** 2026-06-21

**Authors:** Reem Ghonaim, Bahaa A. Hemdan, Hesham R. El-Seedi, Mohamed M. A. Badr, Tarek S. Aysha, Mervat S. El-Sedik, Samar A. El-Kholy, Mehrez E. El-Naggar

**Affiliations:** 1 Department of Chemistry, Faculty of Science, Menoufia University, Shebin El-Kom, 31100107 Egypt; 2 Water Pollution Research Department, Environment and Climate Change Research Institute, National Research Centre, 33 El-Bohouth St., Dokki, Giza, 12622 Egypt; 3 International Research Center for Food Nutrition and Safety, Jiangsu University, Zhenjiang, 212013 China; 4 Department of Biochemistry, Faculty of Pharmacy, Menoufia University, Shebin El-Kom, 31100107 Egypt; 5 Dyeing, Printing and Textile Auxiliaries Department, Textile Research and Technology Institute, National Research Centre, 33 EL-Bohouth St., Dokki, Giza, 12622 Egypt; 6 Pre-Treatment and Finishing of Cellulosic Fabric Department, Textile Research and Technology Institute, National Research Centre, 33 EL-Bohouth St., Dokki, Giza, 12622 Egypt

**Keywords:** Phenothiazine-based dye, Chitosan cryogel, Antimicrobial activity, wound Healing, Biochemistry, Biotechnology, Chemistry, Drug discovery, Microbiology

## Abstract

Cryogels, owing to their low density, high porosity, lightweight structure, and interconnected three-dimensional network, have gained significant attention for biomedical applications. In the present study, a chalcone-based phenothiazine dye derivative (PTZ-chalcone) was successfully synthesized via direct condensation of 2-acetyl-5-bromothiophene with phenothiazine carboxaldehyde derivatives (Ib) using the Kröhnke method in methanolic KOH. The chemical structure of the synthesized compound was confirmed by spectral analysis. The PTZ-chalcone derivative was subsequently incorporated into chitosan (CS) cryogel matrix to fabricate sponge-like biomaterials. The resulting PTZ-chalcone-loaded CS cryogels were systematically characterized and evaluated, with particular emphasis on their antibacterial properties and their potential use as a wound dressing matrix. Likewise, the PTZ-chalcone-loaded CS cryogels displayed strong antibacterial action. In particular, the bactericidal effects of cryogels (CSC2 and CSC3) were demonstrated against *Escherichia coli* (33.5 ± 0.1 mm), *Pseudomonas aeruginosa* (32.4 ± 0.28 mm), *Acinetobacter baumannii* (31.2 ± 0.41 mm), *Staphylococcus aureus* (29.2 ± 0.28 mm), *Streptococcus pyogenes* (28.7 ± 0.35 mm), and *Enterococcus faecalis* (27.5 ± 0.81 mm). The strong antibacterial activity of the PTZ-chalcone compound was validated by bacterial growth curves and protein leakage assays, demonstrating its ability to disrupt bacterial cell membranes. In the Microtox^®^ assay, the cryogels exhibited superior thermal stability and biocompatibility with no harmful effects. Further, the molecular docking analysis showed that PTZ-chalcone has a high binding affinity for proteins ((CDK6 and CDK2), suggesting potential applications in combating bacterial infections and cancer. These results imply that chalcone-loaded CS cryogels are efficient multifunctional biomaterials with great potential for woundhealing applications.

**Supplementary Information:**

The online version contains supplementary material available at 10.1038/s41598-026-55319-w.

## Introduction

Skin is the largest functional organ; its roles include sweating, vitamin D synthesis, and protection of the body from external factors such as infections, toxins, and UV radiation^ 1^. However, some fatal issues, e.g., skin wounds, restrain the skin functionality and performance. A wound is a disruption of the anatomical and cellular continuity of tissue that occurs due to internal factors (Surgical operations) or external factors (accidents). A wound provides a favorable microenvironment for infection by various pathogens, which release virulence factors to enhance their adhesion and invasion, resulting in failure of the healing process^ 2^. Additionally, non-healing wounds have been reported as a risk factor for skin cancer development. This is due to similarities between the tumor stroma and wounded skin, including fibroblasts, blood vessels, and inflammatory cells, which may promote cancer cell proliferation^ 3,4^. Thus, skin injury is severe, and its healing and rehabilitation are urgent to preserve the body’s health.

Wound healing is a physiological process aimed at restoring damaged tissues and preventing infection through a sequential set of steps involving hemostasis, inflammation, proliferation, and remodeling^ 5,6^. For effective wound healing, proper dressing materials are required. The ideal wound dressing should be hypoallergenic, prevent microbial infection, improve cell migration, and enhance angiogenesis and tissue remodeling ^7^. In addition to these requirements, dressing materials should be non-toxic, biocompatible, and easy to remove after healing. It is quite challenging to develop dressings that meet such numerous requirements and perform consistently under different wound and patient conditions. Modern biomaterial dressings, such as bio-based cryogels, hydrogels, films, and nanofibers, have recently shown remarkable benefits in more challenging scenarios, as they are functionalized to accelerate the healing process and prevent microbial infection ^8^. A blended chitosan (CS)-glycerol solution forms elastic, biodegradable, and biocompatible films. The prepared films serve as suitable dressing materials with enhanced healing efficiency and as drug carriers for water-soluble and water-insoluble antibacterial drugs, with sustained release. This blend showed greater elasticity and stronger antibacterial activity against *Escherichia coli* and *Staphylococcus aureus*. 

On the other hand, CS cryogels exhibited additional properties suitable for wound healing compared with their films, nanofibers, and membranes. This is mainly attributed to their interconnected porous structure, which mimics the extracellular matrix and thereby promotes healing. Cryogels are three-dimensional (3D), sponge-like structures that have attracted considerable attention in various fields, owing to their exceptional features, i.e., high porosity, high surface area, flexibility, an interconnected network, high encapsulation efficiency, swelling-deswelling behavior, and water-retaining capabilities^ 9–11^. Furthermore, cryogel was synthesized via a facile, rapid freeze-drying technique, thereby facilitating its application at both laboratory and industrial scales. Bölgen et al.^ 12^ stated that they prepared 3D porous cryogels based on CS and *Hypericum perforatum* oil to develop potential wound-dressing materials that promote both new tissue formation and wound healing. The resulting cryogels were characterized by high porosity, elasticity, suitability for exudative wounds, including diabetic wounds, and satisfactory antimicrobial activity against *E. coli* and *Legionella pneumophila*. Based on CS, citric acid, and silver nanoparticles, Cao et al. ^13^ prepared CS/CA/Ag cryogels with shape-memory behavior, antibacterial activity, high adhesion, blood-absorption capacity, and satisfactory flexibility, making them suitable as hemostatic agents for preventing severe bleeding from damaged skin.

Thus, the present study focuses on the preparation of CS cryogels enriched with Phenothiazine–chalcone (PTZ-chalcone) to design biologically functionalized scaffolds as potential wound dressings. This formulation combines a synthetic bioactive agent with a natural polymer to synergistically meet the requirements for effective wound healing. Because the prepared PTZ-chalcone possesses exceptional antimicrobial properties, it may also promote wound healing. In addition to itbiocompatibility, non-toxicity, biodegradability, and suitability for biomedical applications, these components were assembled into cryogels that mimic extracellular matrix structure, provide a wet environment, and absorb wound exudates^ 14–16^.

To our knowledge, this is the first report of encapsulating PTZ-chalcone within CS matrix via cryogelation to fabricate porous scaffolds for wound dressing. CS is a polysaccharide derived from the deacetylation of chitin, the world’s second-most-prevalent natural polymer. Its structure comprises glucosamine and *N*-acetylglucosamine residues linked by a 1,4-*β*-linkage ^17–19^. CS is a unique biopolymer with desirable properties, including biodegradability and biocompatibility, as well as pharmacological activities such as antibacterial and anti-inflammatory effects, hemostatic properties, and the ability to promote skin regeneration. Additionally, CS exhibits excellent water absorption and retention capabilities ^20^. As a cationic biodegradable polymer, CS tends to interact strongly with the negatively charged skin surface   ^21,22^. 

Moreover, the term chalcone is a generic term for compounds with a 1,3-diphenylprop-2-en-1-one skeleton. It can be found in tea leaves, vegetables, and fruits. Chalcones can be synthesized via Aldol or Claisen-Schmidt condensation reactions^ 14^. Chalcones piqued researchers’ interest because of their versatility in skeleton modification. Those altered chalcones to exhibit several biological properties, including antimicrobial, anti-inflammatory, anticancer, antioxidant, antidiabetic, antileishmanial, and antimalarial activities ^23^. Phenothiazine is a tricyclic compound that contains nitrogen and sulfur. Its derivatives exhibit highly flexible biological actions. Phenothiazine appears in 16 FDA-approved pharmaceutical compounds. Phenothiazine derivatives have been reported to exhibit antibacterial, antifungal, antiviral, anti-inflammatory, antiparasitic, and multidrug-resistance reversal activities ^24^. Specifically, Phenothiazine-based chalcones have attracted considerable attention due to their highly efficient antibacterial activities ^14,25,26^.

In this context, a phenothiazine-conjugated chalcone was synthesized and characterized by NMR and mass spectrometry. The synthesized 1-(5-bromothiophen-2-yl)-3-(10-hexyl-10 H-phenothiazin-3-yl)prop-2-en-1-one was loaded into CS at varying concentrations and freeze-dried to form cryogels. The synthesized cryogels were assessed for their antimicrobial and wound-healing properties. Additionally, an in silico molecular docking study was performed to assess the anticipated antibacterial activity of PTZ-chalcone.

## Materials and methods

### Materials

Phenothiazine (98%), 1-bromohexane, and 2-acetyl-5-bromothiophene were acquired from Sigma-Aldrich (Germany). All solvents (dimethylformamide (DMF), acetone, ethanol, methanol, 1,4-dioxane, acetonitrile, and n-hexane) were used as supplied without further purification and are of analytical grade. Chitosan (CS) was purchased from Oxford Laboratory, India. Glutaraldehyde (25%) with analytical grade purchased from LOBA CHEMIE, India. The progress of the reactions and product formation was characterized by Thin-layer Chromatography (TLC) using Silica gel plates (Merck, Kieselgel 60 F254). FTIR transmittance spectrum of PTZ-chalcone compound was obtained using Vertex 70 FTIR spectrometer from Bruker Optik GmbH, Germany, using KBr pellet method in the spectral range of 4000 –400 cm^–1^ with the resolution of 4 cm^-1^, averaging 50 scans per sample. Proton and carbon nuclear magnetic resonance spectra (^1^H and ^13^C NMR) were obtained using DMSO-d_6_ as solvent and TMS (δ 0 ppm) as internal reference on a Bruker DMX-400 operating at 400 and 101 MHz at the time of the study. Mass Spectrometry (MS) data were obtained using an LC–MS system with an electrospray ionization (ESI) source operated in positive ion mode (XEVO TQD triple quadrupole mass spectrometer).

### Methods

#### Synthetic routes of PTZ-chalcone (1-(5-bromothiophen-2-yl)-3-(10-hexyl-10 H-phenothiazin-3-yl) prop-2-en-1-one)

**Scheme 1:**
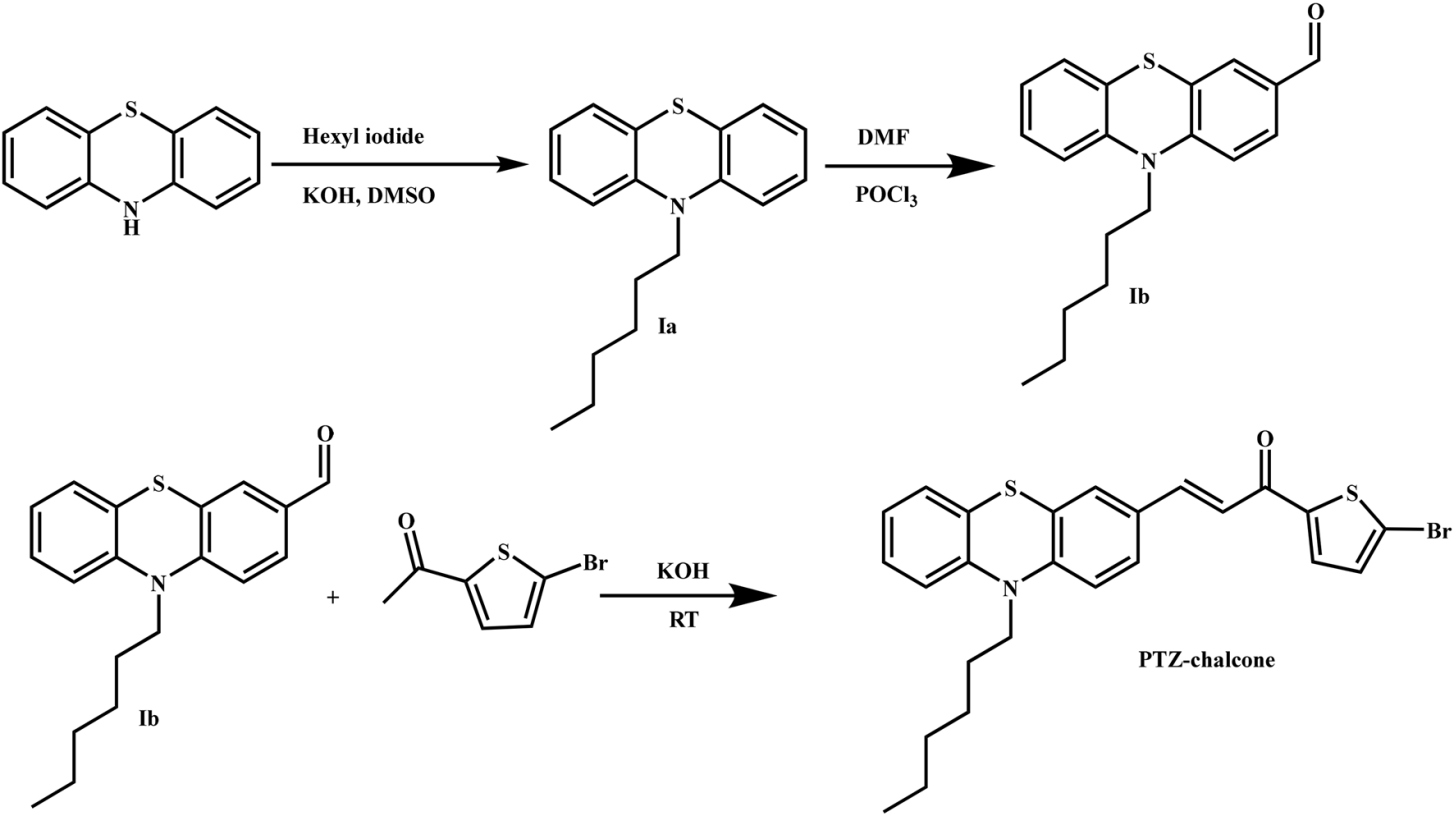
The synthetic procedure of PTZ-chalcone.

The *N-alkylated* phenothiazine **Ia** and its carboxaldehyde derivative **Ib** in Scheme 1 were prepared as previously described ^27–29^.

#### 1-(5-bromothiophen-2-yl)-3-(10-hexyl-10 H-phenothiazin-3-yl) prop-2-en-1-one

In a 100 mL round-bottom flask, 2-acetyl-5-bromothiophene (0.41 g, 2.0 mmol) and PTZ (Ib) (0.60 g, 2.0 mmol) were dissolved in 50 mL of methanol, and the mixture was stirred at room temperature and 30% KOH (10 mL) was added to the mixture dropwise over 20 min. The mixture was stirred at ambient temperature for 8 h, and the reaction progress was monitored by TLC. And the reaction mixture became reddish over time. Subsequently, the reddish mixture was neutralized with 10% HCl, the precipitate was filtered off, washed several times with water, and dried at 40 °C. The final purified product was obtained by recrystallization in ethanol, affording 0.6 g (60% yield).

**FTIR (KBr, cm**^–1^): 3076–3057 cm^–1^ (aromatic C–H), 2952–2852 cm^–1^ (aliphatic C–H), 1670 cm^–1^ (C = O, α,β-unsaturated ketone), 1600–1500 cm^–1^ (C = C, aromatic and olefinic), 1350–1250 cm^–1^ (C–N), 750–700 cm^–1^ (C–S), 650–500 cm^–1^ (C–Br).

**ESI mass analysis**:- m/z (calculated) = 497.05, m/z (found) = 498.0796 [M + H]^+ ^90%, 500.0351 [M+ 3H]^+^ 100%.

^**1**^**H NMR (DMSO-*d***_*6*_, **500 MHz)**: δ(ppm) 0.77 (t, *J* = 6.9 Hz, 3H, CH_3_), 1.18–1.22 (m, 4H, 2CH_2_), 1.34 (m, 2H, CH_2_), 1.63 (m, 2H, CH_2_), 3.85 (t, *J* = 7.0 Hz, 2H, CH_2_), 6.93 (t, *J* = 7.4 Hz, 1H, CH aromatic), 6.98 (d, *J* = 3.5 Hz, 1H, CH aromatic), 7.00 (d, *J* = 3.2 Hz, 1H, CH aromatic), 7.11 (d, *J* = 7.7, 1.6 Hz, 1H, CH aromatic), 7.17 (t, *J* = 7.7 Hz, 1H, chalcone CH), 7.42 (d, *J* = 4.1 Hz, 1H, CH aromatic), 7.58–7.64 (m, 2H, CH aromatic), 7.67 (s, 1H, CH aromatic), 7.70 (d, *J* = 2.1 Hz, 1H, CH aromatic), 8.13 (d, *J* = 4.1 Hz, 1H, chalcone CH).

^**13**^**C NMR (101 MHz**,** DMSO-d**_**6**_**)**:- δ 191.20 (C = O), (180.93, 148.14, 147.38, 146.01, 144.02, 143.25, 134.34, 132.90, 130.26, 129.22, 128.21, 127.63, 127.26, 124.25, 123.48, 123.16, 122.43, 119.09, 116.57, 116.02) represent the aromatic and *α,β* unsaturated chalacone carbon, 47.20(CH_2_), 31.27(CH_2_), 26.59(CH_2_), 26.22(CH_2_), 22.52 (CH_2_), 14.27 (CH_3_).

### Assessment of antibacterial activities of PTZ chalcone compound

#### Bacterial strains and inoculum preparation

Six clinically relevant skin pathogenic bacterial species were selected to evaluate the antibacterial potential of the PTZ-chalcone compound. Gram-negative bacteria included *Escherichia coli*, *Pseudomonas aeruginosa*, and *Acinetobacter baumannii*, while Gram-positive bacteria comprised *Staphylococcus aureus*, *Streptococcus pyogenes*, and *Enterococcus faecalis*. Lyophilized cultures were revived in tryptic soy broth (TSB) and incubated aerobically at 37 °C for 18–24 h. The resulting cultures were centrifuged at 7000 rpm for 15 min to obtain bacterial pellets, which were washed and resuspended in sterile phosphate-buffered saline (PBS). Bacterial suspensions were standardized spectrophotometrically at 600 nm to match the 0.5 McFarland turbidity standard, corresponding to approximately 1.5 × 10^8^ CFU/mL. A stock solution of PTZ-chalcone (10 mg/mL) was prepared and sonicated for 15 min prior to use to ensure uniform dispersion. All assays were conducted in triplicate and repeated on three independent days ^30^.

#### Determining the inhibition zone through agar diffusion assay

Antibacterial activity was preliminarily screened using the agar well diffusion technique. Standardized bacterial suspensions were uniformly spread on Mueller–Hinton agar (MHA) plates using sterile swabs. Circular wells (6 mm diameter) were aseptically punched into the agar surface and filled with 50 µL of the PTZ-chalcone solution. Ciprofloxacin (50 µg/mL) served as the positive control, and sterile distilled water as the negative control. Plates were incubated at 37 °C for 18–24 h. After incubation, the diameters of inhibition zones were measured using a digital Vernier caliper and expressed in mm.

#### Evaluating the viability of bacterial cells

Bacterial cell viability and the minimum inhibitory concentration (MIC) of the PTZ-chalcone compound were evaluated using a macrodilution assay. Standardized suspensions of *E. coli*,* P. aeruginosa*,* A. baumannii*,* S. aureus*,* Str. pyogenes*, and *E. faecalis* (1.5 × 10⁶ CFU/mL) were prepared and transferred into sterile tubes containing 10 mL of sterile medium. The bacterial suspensions were exposed to increasing concentrations of PTZ-chalcone (25, 50, 100, 150, and 300 mg/mL)^ 31^. At predetermined exposure times (0, 5, 10, 20, and 30 min), aliquots were collected, serially diluted, and plated onto agar media. The bactericidal activity of the compound was determined by comparing viable counts with those of untreated control cultures containing bacteria ^32^.

#### Evaluation of physiological alterations in bacterial cells

Physiological responses of the tested bacterial strains (*E. coli*,* P. aeruginosa*,* A. baumannii*,* S. aureus*,* Str. pyogenes*, and *E. faecalis*) to PTZ-chalcone exposure were investigated using growth kinetics analysis and intracellular protein leakage measurements.

### Growth kinetics assay

To monitor bacterial growth, 100 µL of each standardized bacterial suspension was inoculated into 50 mL of sterile tryptic soy broth (TSB). One culture served as an untreated control, while a second culture contained PTZ-chalcone at its effective concentration. Cultures were incubated at 37 °C in a shaking incubator (200 rpm). At 3 h intervals over a 24 h period, 1 mL samples were withdrawn, and bacterial growth was assessed by measuring optical density at 600 nm (OD₆₀₀) using a UV–visible spectrophotometer ^33^.

### Intracellular protein leakage assay

Membrane damage induced by PTZ-chalcone was evaluated by quantifying the release of intracellular proteins using the Bradford colorimetric method ^34^. After treatment, 2 mL aliquots of bacterial cultures were centrifuged at 5000 rpm for 10 min, and the supernatants were collected for analysis. Each supernatant sample was mixed with 800 µL Bradford reagent and incubated in the dark at 37 °C for 10 min. Absorbance was then measured at 595 nm. Protein concentration was determined using a bovine serum albumin (BSA) standard calibration curve.

### Preparation of chitosan cryogel loaded with different concentrations of PTZ-chalcone

Chitosan (Cs) solution was prepared by dissolving 1.5 g in 100 mL of acetic acid solution (1%) under mechanical stirring for 1 h. Meanwhile, the PTZ-chalcone solution was prepared by dissolving 1 g in 40 mL of acetone. For cryogel preparation, CS and PTZ-chalcone solutions were blended as tabulated in Table 1, maintaining a total volume of 30 mL. The blending of CS and PTZ-chalcone solutions was performed under vigorous stirring for 15 min. Then, 0.25 mL of glutaraldehyde was added to each mixtures, and stirred for an additional 5 min. Afterward, the blended solutions were sonicated for 30 min to remove the air bubbles. Finally, the sonicated solutions were placed in a freezer at -20 °C for 24 h and dried using freeze-drying-instrument (ZIRBUS, Germany) at − 60 °C and 0.1 atm.

**Table 1:** The volumes of CS and PTZ-chalcone for cryogel formation.

Samples codes	CS solution (mL)	PTZ-chalcone solution (mL)
**CSC0**	30	0
**CSC1**	28	2
**CSC2**	26	4
**CSC3**	24	6

### Characterization of CSC0, CSC1, CSC2 and CSC3

Surface morphological features and chemical composition of the as-prepared cryogel were examined using a scanning electron microscope (SEM; TESCAN, VEGA 3, Czech Republic). FTIR (Bruker VERTEX 80, Germany) was used to assess the functional groups of the cryogel between 4000 and 400 cm^–1^. The thermogravimetric analyses (TGA) were carried out using a PerkinElmer TGA4000. TGA of the cryogels was obtained by heating the samples at a temperature range (50–600 ˚C) at a rate of 0.1 to 200 ˚C/min in a nitrogen atmosphere. Surface area and pore-size distribution of the CS cryogels (CSC0 and CSC3) were measured using an ASAP 2020 specific surface area and porosity analyzer (Micrometrics Instrument Corporation) after degassing the samples for 24 h at 100 °C.

### Antibacterial activity of cryogel composites

The bactericidal activity of the prepared cryogels was evaluated using a direct-contact assay. Sterile cryogel samples (5 × 5 mm) were placed in sterile tubes containing fresh tryptic soy broth (TSB). A 10 µL aliquot of a freshly prepared bacterial suspension was added to each tube. The tubes were incubated at 37 °C with shaking at 150 rpm for varying contact periods (0.5–6 h). At the designated time points, aliquots were collected before and after exposure to the cryogels, serially diluted in sterile saline, and plated using the pour plate method. Following incubation at 37 °C for 24 h, viable bacterial cells were enumerated and expressed as CFU/mL. The following formula was used to calculate the bacterial log CFU reduction ^35^:

$$Log~CFU~reduction=Log~CFU{~_{control}} - Log~CFU{~_{after~treatment}}$$

### The inhibition of bacterial biofilm development

A static adhesion model was used to evaluate the antibiofilm activity of the cryogel formulations (CSC0, CSC1, CSC2, and CSC3). The sterile cryogel swatches (5 × 5 mm) were placed on 12-well plates containing 10 mL of PBS. To enable bacterial adhesion and biofilm development on the cryogel surface, 100 µL of a 24 h bacterial culture was added to every well, and the wells were then incubated at 37 °C. Following incubation, the adhered biofilm was carefully scraped from the cryogel surface to mechanically separate it. The collected suspensions were serially diluted and plated onto agar medium to determine viable cell counts. The number of attached bacteria was expressed as CFU/cm^2^.

### Biocompatibility and toxicity assessment

The biological safety of the PTZ-chalcone compound and the prepared cryogel formulations was evaluated using a Microtox^®^ Model 500 analyzer (Modern Water Inc., New Castle, DE, USA). This assay measures the inhibition of bioluminescence emitted by the marine bacterium *Vibrio fischeri* following exposure to the tested samples. The reduction in light emission was used to determine the toxicological profile of each formulation, thereby assessing their suitability for potential biomedical applications. The Microtox^®^ assay was conducted under standardized conditions, including appropriate sample dilution and control measurements, to minimize potential interference from turbidity or sample coloration. Accordingly, the obtained EC_50_ % values are considered to accurately reflect the biological response of *V. fischeri* rather than analytical objects ^36^.

### Molecular docking

The three-dimensional structure of PTZ-chalcone was constructed and energy-minimized in Avogadro (version 1.2.0) using the MMFF94 force field. Protein targets, including mTOR, CDK2, CDK6, and DNA gyrase B from *Str. pyogenes* and *P. aeruginosa* were retrieved from structural databases. Prior to docking, protein structures were prepared using AutoDock Tools (version 1.5.7) by removing crystallographic water molecules, adding polar hydrogens, and assigning Gasteiger charges. Binding pockets were identified using the CB-DOCK2 server^ 37^. Docking simulations were performed using AutoDock Vina to predict binding modes and calculate binding affinities (ΔG) ^38^. The resulting ligand–protein interactions were visualized and analyzed using BIOVIA Discovery Studio Visualizer^ 39^.

### The ADMET evaluation

Pharmacokinetic behavior and toxicity characteristics of PTZ-chalcone were predicted using the ADMETlab 2.0 online platform. This computational tool was employed to estimate absorption, distribution, metabolism, excretion, and toxicity parameters ^40^.

### Statistical analysis

The observational data were analyzed using two-way ANOVA. The findings were reported as mean ± SD, with significance defined as *P* < 0.05. The significance norm for the experimental trials was established.

## Results and discussion

### Synthesis of PTZ-chalcone

Synthetic routes of PTZ-chalcone (1-(5-bromothiophen-2-yl)-3-(10-hexyl-10 H-phenothiazin-3-yl) prop-2-en-1-one) were summarized in Scheme 1. The synthesis of PTZ-chalcone starts with the direct alkylation of phenothiazine, affording *N*-hexylphenothiazine derivative (**Ia**), followed by Vilsmeier formylation to give 10-hexyl-10 H-phenothiazine-3-carbaldehyde (**Ib**), as previously reported^ 41^. The PTZ chalcone was formed in good reaction yield by direct condensation of 2-acetyl-5-bromothiophene with the phenothiazine carboxaldehyde derivatives (**Ib**) using the Kröhnke method in the presence of methanolic-KOH reagents.

The FTIR spectra using the KBr method were investigated to confirm the functional group of the prepared phenothiazine based chalcone (Fig. S1). Stretch medium characteristic bands appear at 3076–3057 cm^–1^, corresponding to the aromatic (= C-H) phenothiazine and thiophene rings, while aliphatic (–C-H-) related to the terminal hexyl chain appears as a medium stretch band at 2852–2952 cm^–1^. The *α*,*β* unsaturated carbonyl group appears as a strong stretch band at 1670 cm^–1^. The olefinic (–C = C-) of chalcone appears at 1522 cm^–1^, the (-C-N-) appears at 1463 cm^–1^ while (–C-S and –C-Br-) appear at 745 and 664 cm^–1^, respectively.

The ESI mass spectra showed a significant peak at 498.0796 corresponding to the +ve ion, indicating the formation of PTZ chalcone, as shown in Fig. S2.

NMR spectra was used to prove the structure of PTZ-chalcone. The ^1^ HNMR spectra showed characteristic peaks at 0.76–3.85 δ corresponding to the *N*-alkylated protons, the terminal methyl proton in hexyl alkane appears as triplet at 0.76–0.79 δ and four of the methylene protons appears as multiplets in the range of 1.18–1.63 δ while the methylene protons attached to the nitrogen in the phenothiazine ring appears as triplet at 3.85 δ. The protons of *α*,*β* unsaturated keton (chalcone) appear at 7.17 and 8.13 δ as doublet. The aromatic protons show a huge overlap and appear in the range of 6.93–7.70 δ as shown in Fig. S3. Two triplet aromatic protons corresponding to the phenothiazine ring appear at 6.93 and 7.17 δ, while the other aromatic protons appear as a doublet and a singlet, which were recorded at 6.98, 7.00, 7.11, 7.42, 7.58, 7.64, 7.67, and 7.70 δ, as shown in Fig. S3.

The ^13^C NMR spectrum showed all the corresponding carbon peaks of PTZ-chalcone; the terminal methyl group carbon in the hexyl chain appears at 14.27 δ, while the other methylene carbon appears at 22.52, 26.22, 26.59, 3127, and 47.20 δ. The carbonyle carbon appears at 191.20 as shown in Fig. S4.

### Antibacterial properties of PTZ chalcone compound

**Fig. 1:**
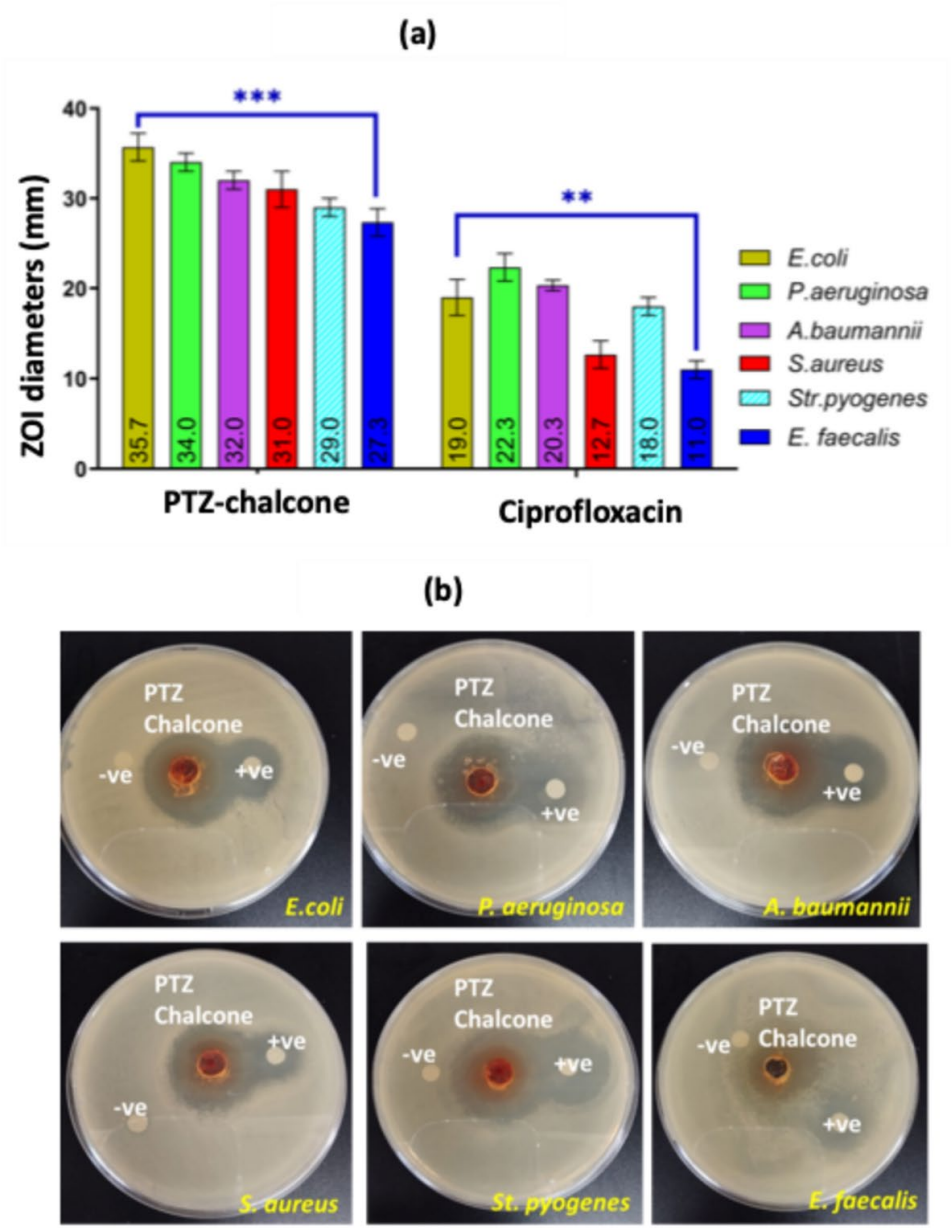
(a) Antimicrobial activity of the PTZ-chalcone compound against six pathogenic bacteria using the agar well diffusion assay. (b) Representative photographic images of the agar plates. The zones of inhibition (ZOI) are expressed as diameters (mm) and presented as mean ± SD. Statistical significance is indicated as follows: *** *P* < 0.001 (high significance) and ** *P* < 0.01 (moderate significance) compared to the tested bacterial strains.

According to the findings shown in Fig. 1 (a, b), an agar well diffusion assay was used to assess the antibacterial activity of a PTZ-chalcone compound against six prevalent cutaneous bacterial infections. The activity of Ciprofloxacin, which served as a positive control, is contrasted with the findings. The PTZ-chalcone compounds showed the most potent antibacterial activity against *E. coli*, with a zone of inhibition (ZOI) of 35.7 mm. Similarly, *P. aeruginosa*,* A. baumannii*,* S. aureus*, and *Str. pyogenes* and *E. faecalis* had ZOI of 34.0, 32.0, 31.0, 29.0, and 27.3 mm, respectively.

Although the ZOI diameters of the positive control, ciprofloxacin, were often smaller than those of the PTZ-chalcone compound, the PTZ-chalcone compound still demonstrated antibacterial efficacy against the tested microorganisms. Where the ZOI diameters of *E.coli*,* P. aeruginosa*,* A. baumannii*,* S. aureus*, *Str. pyogenes*, and *E. faecalis* were 19.0, 22.3, 20.3, 12.7, 18.0, and 11.0 mm, respectively. According to the statistical analysis, the PTZ-chalcone compound and Ciprofloxacin showed statistically significant changes in ZOI diameters, with P-values < 0.0001 for the greater effects and *P* < 0.0016 for the moderate effects. These findings suggest that the PTZ-chalcone compound may have antibacterial activity, particularly against common skin bacterial infections such as *E. coli*,* P. aeruginosa*,* A. baumannii*, and *S. aureus*^ 42^.

The PTZ-chalcone compound exhibits promising antibacterial activity and can be used to treat skin infections. Common causes of many skin and soft tissue infections, including cellulitis, impetigo, folliculitis, and wound infections, are *E. coli*,* P. aeruginosa*,* A. baumannii*, and *S. aureus*. The potent antibacterial action of the PTZ-chalcone compound was demonstrated by extensive inhibition zones against these pathogens, indicating its potential. In particular, it was effective against *E. coli*-related skin infections, which may arise in immunocompromised people or in the setting of wound infections, explained by the powerful inhibitory activity reported against *E.coli* (ZOI of 35.7 mm). The compound exhibits moderate-to-strong activity against *P. aeruginosa*,* A. baumannii*, and *S. aureus*. These opportunistic pathogens pose significant challenges in clinical settings due to increasing antibiotic resistance. These findings suggest that the compound can be useful in managing infections caused by these pathogens. Although the compound’s activity against *Str. pyogenes* and *E. faecalis* were somewhat lower, the inhibition zones indicate that the compound may also be helpful in treating infections caused by these organisms, possibly in conjunction with other antimicrobial agents ^42^.

### Estimation of bacterial cell viability

**Fig. 2:**
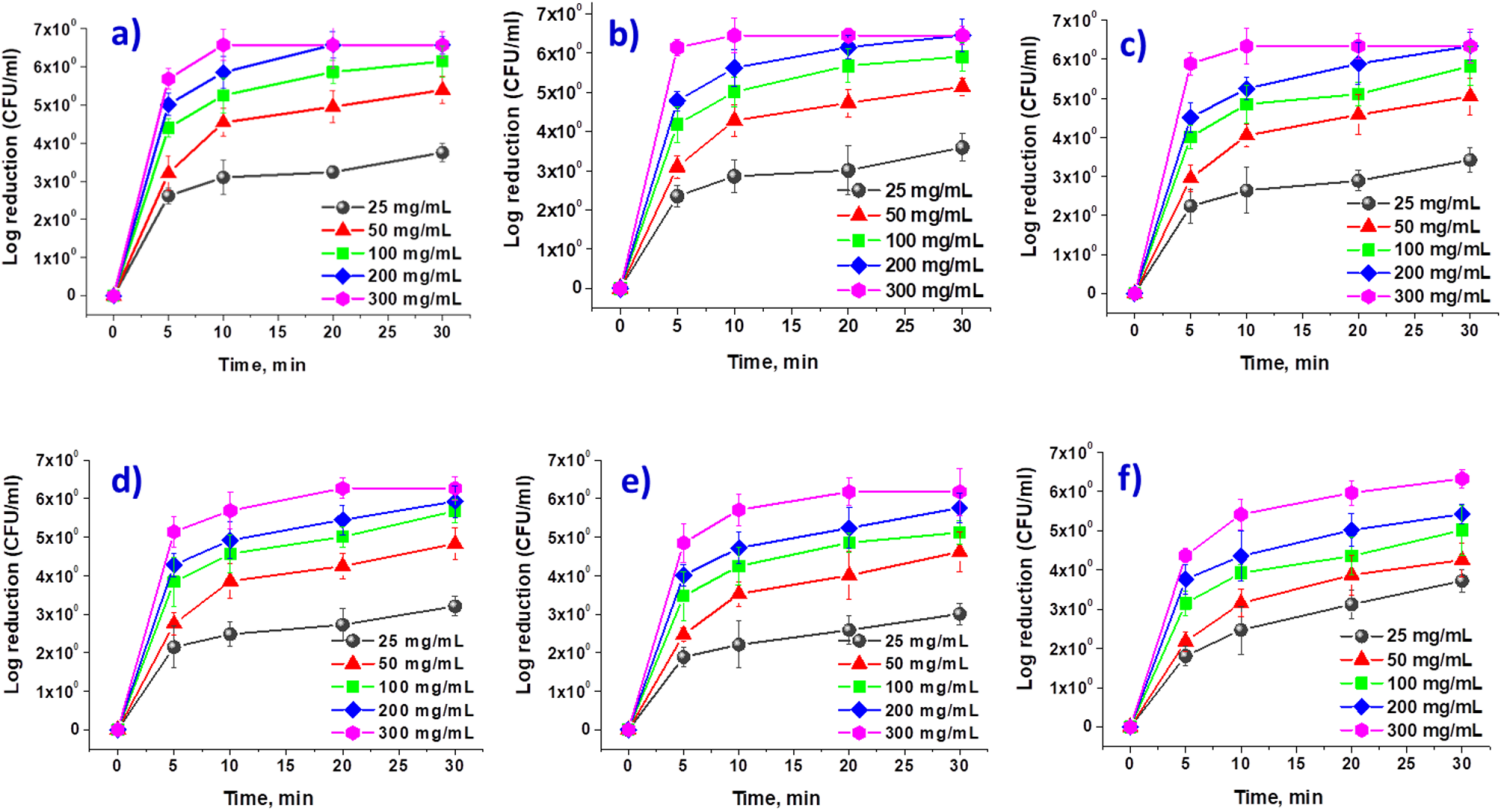
The rate of log-CFU reduction of certain bacterial strains (a) *E.coli*, (b) *P. aeruginosa* (c) *A. baumannii*, (d) *S. aureus*, (e) *Str. pyogenes*, and (f) *E. faecalis* after being subjected to PTZ- chalcone compound at different dosages ranging from 25–300 mg/mL during the various exposure times ranging from 0–30 min.

The log bacterial reduction rate and the number of persistent bacterial cells (*E.coli*,* P. aeruginosa*,* A. baumannii*,* S. aureus*,* Str. pyogenes*, and *E. faecalis*) were determined after exposure to different concentrations (25, 50, 100, 200, and 300 mg/mL) of the PTZ-chalcone compound for various durations (0, 5, 10, 20, and 30 min). The colony-forming unit (CFU) technique was used for estimation. The approach was used to determine the optimal dose and duration of the PTZ-chalcone compound to eradicate bacterial populations. The in vitro antibacterial profile of the tested compound showed that the PTZ-chalcone exhibited superior antibacterial efficacy, reducing the 6-log CFU within a brief exposure period (Fig. 2). The results shown in Fig. 2 (a) demonstrate that the total logarithmic count (6-log) of *E.coli* was entirely diminished after being exposed to a concentration of 200 mg/mL for 20 min. Figure 2 (b, c) demonstrates that a 6-log decrease of *P. aeruginosa* and *A. baumannii* was obtained following a 30 min exposure using a 200 mg/mL concentration. In contrast, Gram-positive bacterial species such as *S. aureus*,* Str. pyogenes*, and *E. faecalis* required a greater concentration (300 mg/mL) compared to Gram-negative bacteria (Fig. 2d, e, f). Among the Gram-positive species, 20 min were required for the 6-log CFU of *S. aureus* and *Str. pyogenes* to be completely reduced, whereas *E. faecalis* was eliminated after 30 min.

### Analysis of bacterial growth curves and protein leakage

**Fig. 3:**
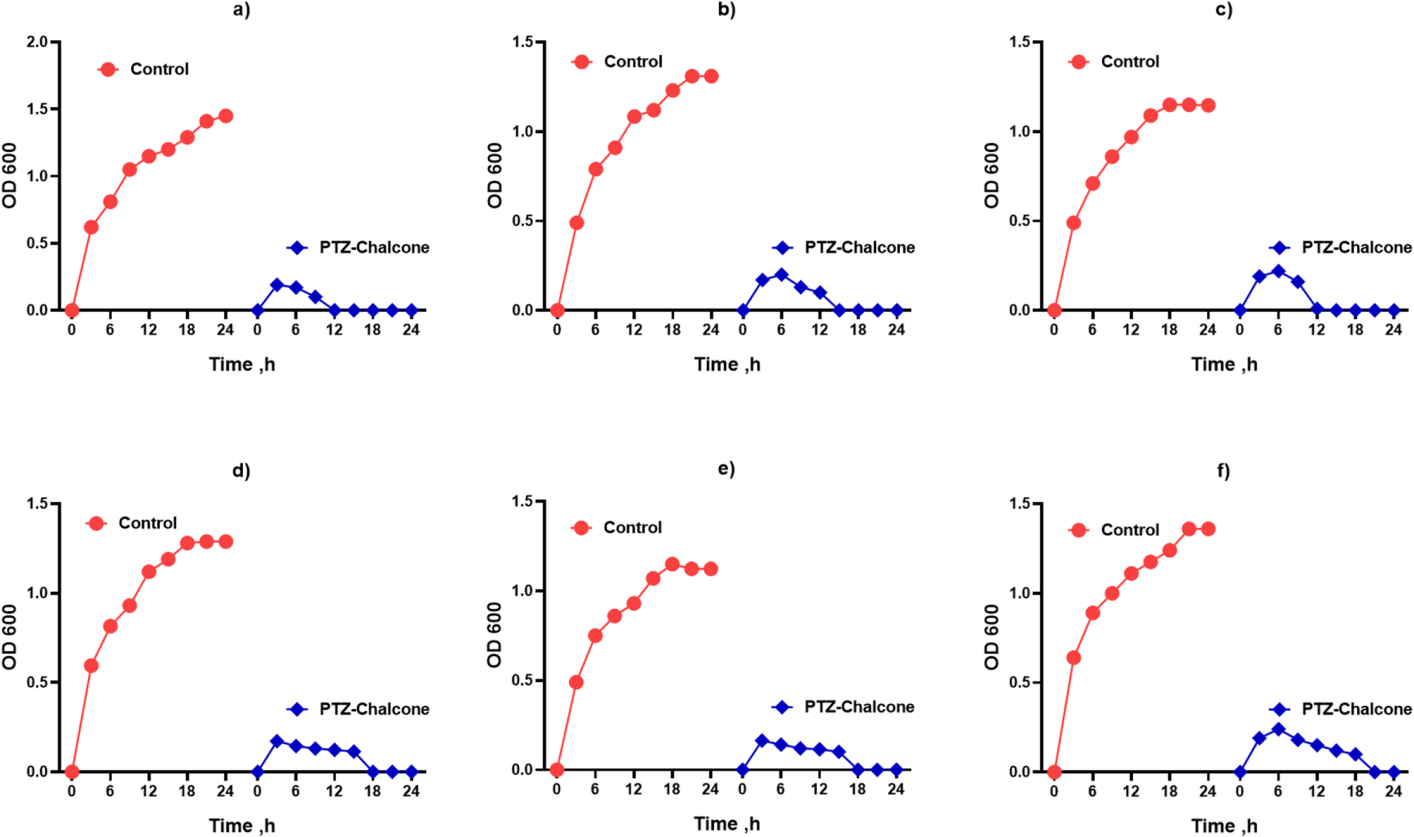
The estimated growth kinetics curves of (a) *E.coli*, (b) *P. aeruginosa* (c) *A. baumannii*, (d) *S. aureus*, (e) *Str. pyogenes*, and (f) *E. faecalis* at normal pattern before and after subjecting to PTZ-chalcone compound.

The bacterial growth curves, displayed in Fig. 3 (a-f), clearly demonstrate that the PTZ-chalcone compound efficiently suppressed bacterial growth at an equivalent concentration of 200 mg/mL. At various time points, the growth of all pathogenic bacteria under investigation was successfully prevented when the PTZ-chalcone was present at 200 µg/mL. More specifically, without going through the traditional growth curve stages, the growth curves of *E. coli*,* P. aeruginosa*,* A. baumannii*,* S. aureus*,* Str. pyogenes* and *E. faecalis* exhibited declines and reached their lowest points at 12, 15, 18, and 21 h, respectively. Conversely, bacterial growth was not inhibited in the absence of the PTZ-chalcone compound in the culture medium, and the cultures reached the stationary phase within 24 h. This suggests that the bacteria could proliferate and reproduce without significant hindrance. In the absence of the PTZ-chalcone compound, the growth curves of all bacterial strains exhibited the characteristic phases of the bacterial growth cycle: lag, log, and stationary. The bacteria’s regular growth pattern suggests they were not exposed to any factors that hindered their development, allowing them to multiply and reach the stationary phase.

**Fig. 4:**
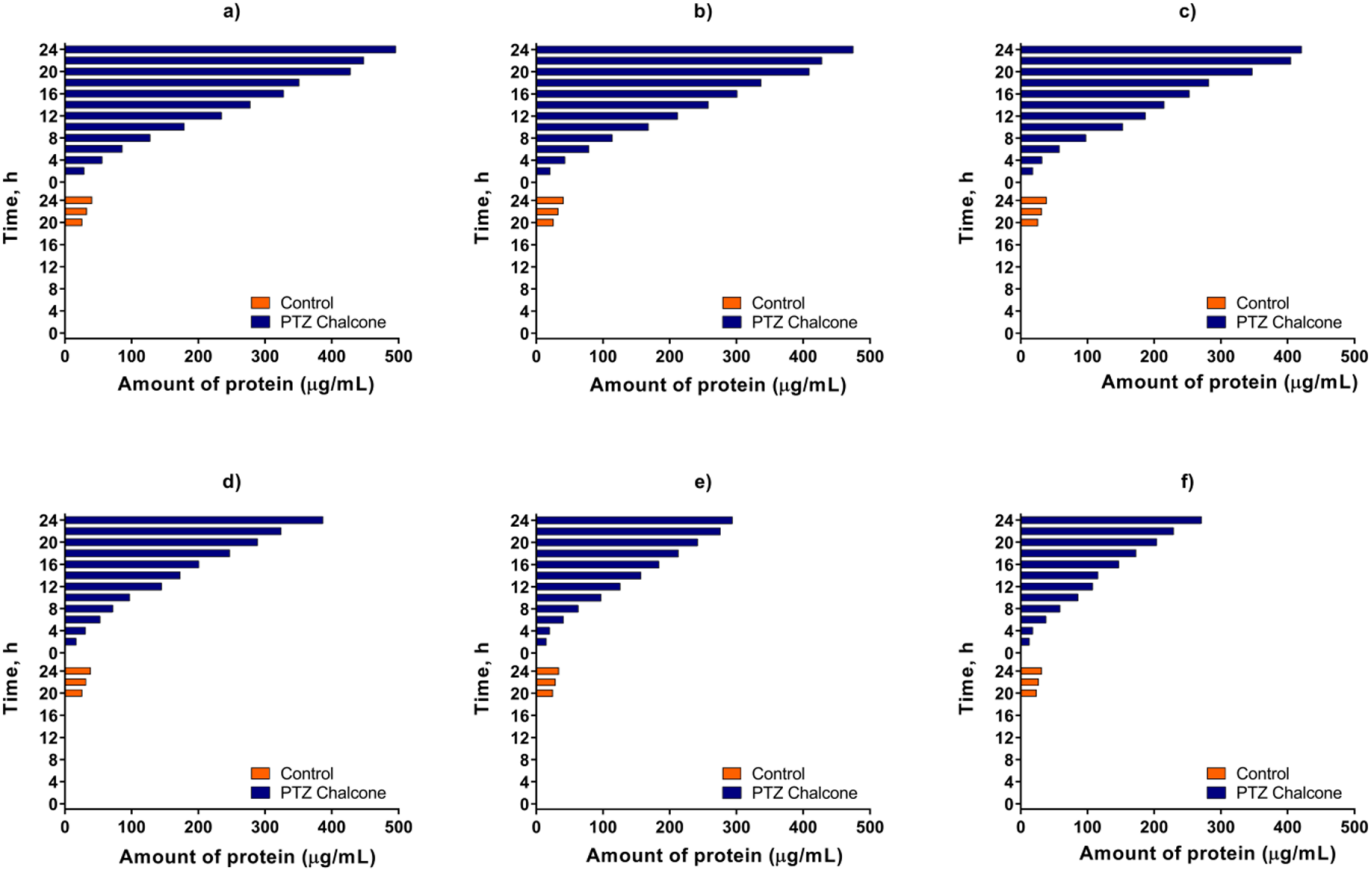
The amount of intracellular protein from damaged bacterial cells of (a) *E.coli*, (b) *P. aeruginosa* (c) *A. baumannii*, (d) *S. aureus*, (e) *Str. pyogenes*, and (f) *E. faecalis* before and after subjecting to PTZ-chalcone compound.

The leakage of intracellular fluids, such as proteins, from different microbes, is a sign of impaired membrane permeability. The amounts of protein released by the investigated bacteria as a function of cellular membrane breakdown are dynamically shown in Fig. 4. The PTZ-chalcone compound (200 mg/mL) was applied to the tested microorganisms for 2–24 h, resulting in a significant increase in the amount of intracellular proteins leaked into the extracellular medium. Treatment of the microbial cells with the PTZ-chalcone resulted in significant protein leakage, whereas the untreated controls showed no detectable protein leakage. For a 2–24 h exposure period, the amount of protein produced from the bacterial and fungal cells rose with the concentration of the PTZ-chalcone molecule. For *E. coli*,* P. aeruginosa*,* A. baumannii*,* S. aureus*,* Str. pyogenes*, and *E. faecalis*, the measured protein leakage was 496, 475, 421, 387, 297, and 271 µg/mL, respectively (Fig. 4a-f). The protein leakage indicates that intracellular proteins are released when the PTZ-chalcone weakens the bacterial cell membrane. The observations show that protein leakage and PTZ-chalcone concentration are dose-dependent, with higher concentrations resulting in greater membrane permeability and protein release. Significant protein leakage from microbial cells is caused by the PTZ-chalcone molecule, which suggests damaged membrane integrity.

The observed suppression of bacterial growth indicates that PTZ- chalcone has a potent antibacterial activity. The compound’s sulfur group is likely to generate reactive oxygen species (ROS), which can disrupt microorganisms’ membranes. Likewise, ROS can damage the lipid bilayer of bacterial cell membranes, increasing permeability and releasing vital biological components. This process compromises the cell membrane’s structural integrity and hinders essential cellular activities, such as respiration and nutrient intake. Consequently, bacterial cells experience a disruption in their ability to maintain internal stability, leading to reduced growth and gradual cell death^43^.

### Morphological assessment of CSC0, CSC1, CSC2 and CSC3

**Fig. 5:**
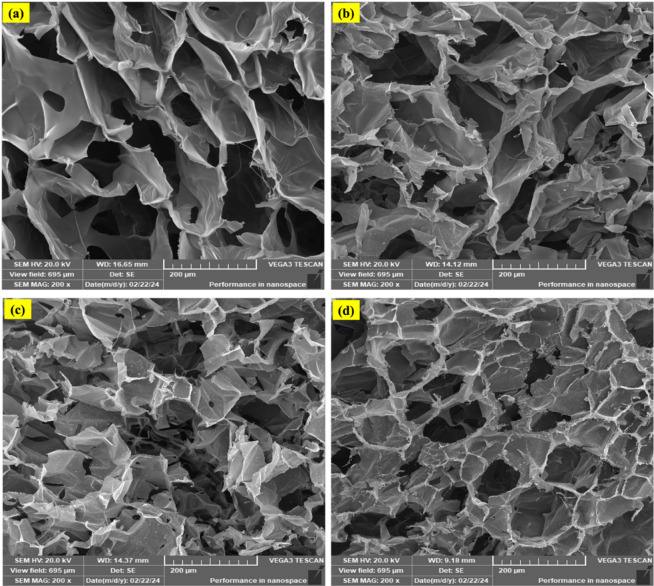
SEM images of CS cryogels, (a) CSC0, (b) CSC1, (c) CSC2, and (d) CSC3.

The surface topography of CSC0, CSC1, CSC2, and CSC3 was investigated using SEM. The SEM micrograph of the pristine CSC0 cryogel is shown in Fig. 5 (a). The SEM of CS cryogels loaded with various concentrations of PTZ-chalcone (2, 4, and 6 mL) is represented in Fig. 5 (b, c, and d), respectively. The pristine CSC0 cryogel exhibited honeycomb morphology with pores ^44,45^. Also, the walls of the CSC0 network are very thin. The addition of PTZ-chalcone at varying concentrations changed the surface morphology of the pristine CSC0 cryogel, providing strong evidence of the incorporation of PTZ-chalcone molecules within the interconnected network of the cryogel. As depicted in Fig. 5 (b), the pores are smaller with thick walls, and PTZ-chalcone molecules begin to deposit within them. Upon further addition (4 and 6 mL), the PTZ-chalcone deposited over the walls, and the pores became much smaller.

### Molecular structure investigation for the prepared cryogels

**Fig. 6:**
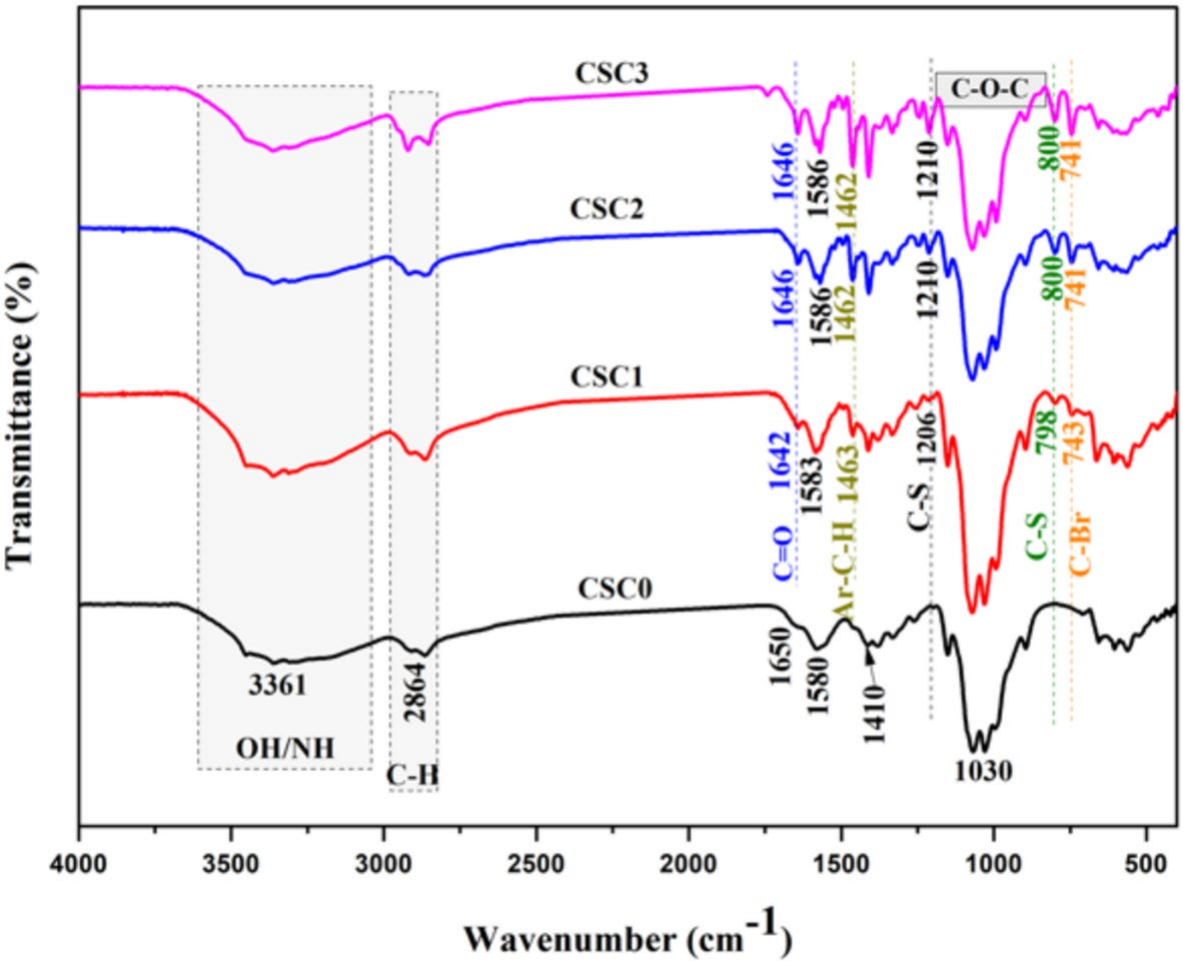
FTIR spectra of CSC0, CSC1, CSC2, and CSC3 cryogels.

Figure 6 displays the FTIR spectra of CS cryogels loaded with different concentrations of PTZ-chalcone (0, 2, 4, and 6 mL), which were designated as CSC0, CSC1, CSC2, and CSC3, respectively. For CSC0 cryogel, its characteristic peaks appeared as follows: at 3361 cm^–1^ (attributed to the stretching vibrations of OH/NH), at 2864 cm^–1^ (asymmetric stretching vibration of CH- (sp^3^)), at 1650 cm^–1^ (stretching vibration of adsorbed water molecules), at 1580 cm^–1^ (vibration mode of amide I), at 1410 cm^–1^ (overlapping bending mode of CH_2_ and of NH_2_), at 1030 cm^–1^ (corresponding to stretching vibration of ether bonds of anhydroglucose unit). The unique peaks of CS present in the spectra indicate that the PTZ is encapsulated with CS cryogel via physical bond formation, and also affirm that the synthesis process didn’t affect the CS’s primary structure. Some variation observed in the CSC1, CSC2, and CSC3 spectra reveals the successful incorporation of PTZ. Whereas, the peak recorded at 1642 cm^–1^ (CSC1), and 1646 cm^–1^ (CSC2 and CSC3) is assigned to C = O stretching vibration of the unsaturated ketone, while the sharp peak at 1583 cm^–1^ (CSC1), and 1586 cm^–1^ (CSC2 and CSC3) is due to the combined vibrations of amide I of CS and C = C of PTZ-chalcone ^46^. Additionally, the C-S vibrations were recorded at 1206 cm^-1^ (CSC1), 1210 cm^–1^ (CSC2 and CSC3),  798 cm^-1^ (CSC1) , and 800 cm^–1 ^^ (CSC2 and CSC3) 47^, and the C-H aromatic bending mode appeared at 1463 cm^-1^ (CSC1), and 1462 cm^–1^ (CSC2 and CSC3). The peak that appeared at 743 cm^–1^ (CSC1), 741 CSC2 and CSC3), 1206 cm^–1^ (CSC1) and 1210 cm^–1^ (CSC2 and CSC3) may be attributed to C-Br.

### Surface area of the prepared cryogels

It is necessary to determine the surface area to characterize the as-prepared cryogels and to evaluate their compound-loading efficiency. Figure S5 illustrates the best results of CSC0 and CSC3. As shown in Fig. S5, the obtained BET isotherm resembles type IV with capillary condensation behavior, ensuring the porous nature of the prepared cryogels ^48^. However, the porosity declined by 33% after PTZ-chalcone incorporation, and the specific surface area decreased from 4.82 m^2^ g^–1^ to 3.62 m^2^ g^–1^, indicating effective encapsulation of PTZ-chalcone molecules within the pores.

### Thermal stability determination of the formed cryogels

**Fig. 7:**
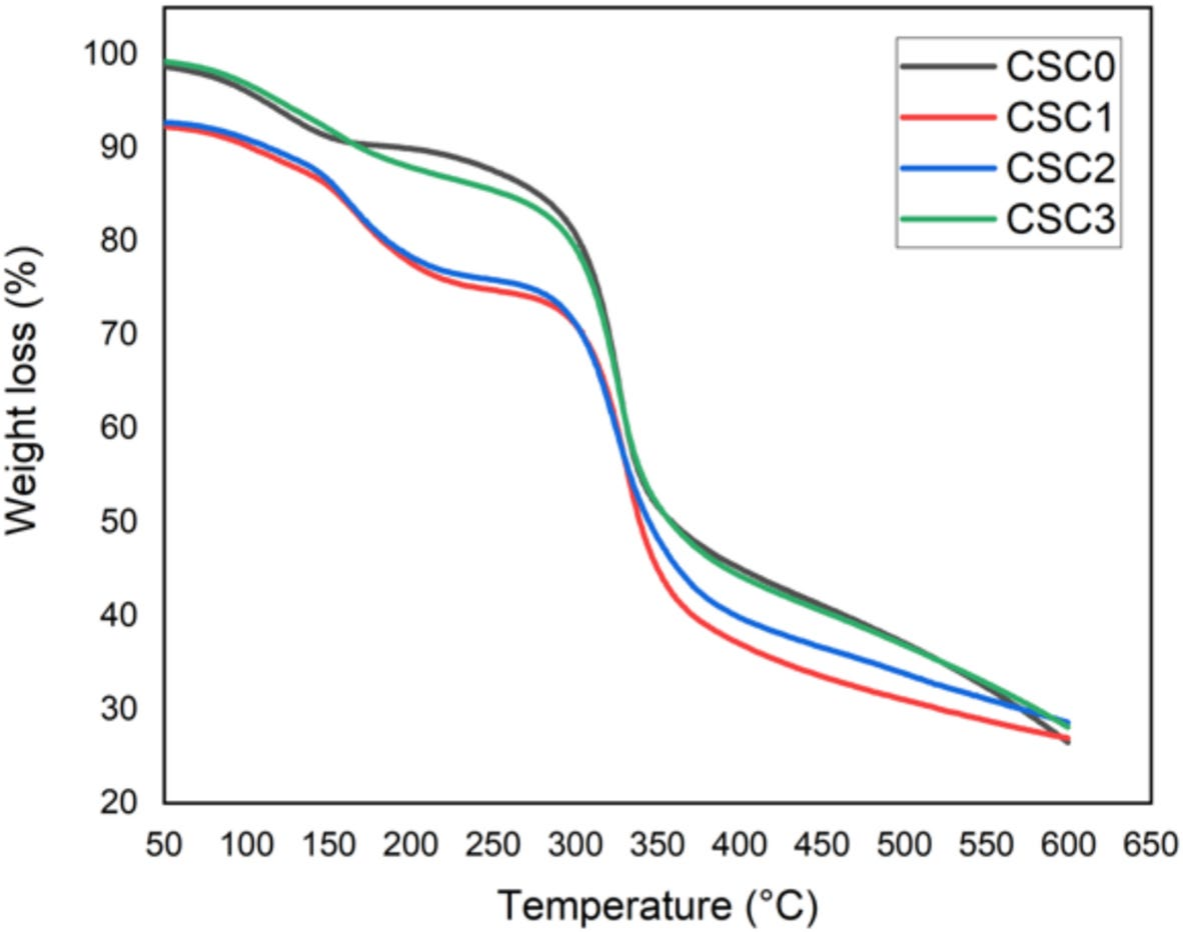
TGA results of the CSC0, CSC1, CSC2, and CSC3.

The thermal stability of PTZ-chalcone-loaded CS cryogels was evaluated using TGA (Fig. 7). The thermal decomposition of the cryogel comprises three stages: the first stage involves liberating adsorbed water molecules on the surface, while the second and third stages are due to polymer degradation. For pristine CSC0 cryogel, water evaporation and residual solvent volatilization (at 50–200 °C) were indicated by 10% weight loss; however, CS chains sharply decomposed, starting at 250 °C, with weight loss reaching 35%; then the CS backbone and/or carbonaceous materials decomposition was observed > 400 °C. Incorporation of the chalcone moiety improves the thermal stability of the as-prepared cryogels because chalcone molecules insert between and adhere to the CS chains via hydrogen bonding, forming a dense, compact, interconnected structure^ 49^. The CSC1 and CSC2 lost about 7% and 6%, respectively, due to moisture removal (50–100 °C), while CS chain disintegration was observed at 300–600 °C, with weight loss reaching 39% and 35%, respectively, after which the plateau is reached. However, the thermal stability of CSC3 decreased slightly due to its aggregation at higher PTZ-chalcone concentrations ^50^.

### Assessment of the bactericidal effect of cryogel composites

#### Antibacterial effect of prepared cryogel composites

For various biomedical applications, the prepared cryogel materials have attracted significant attention, especially in wound care ^51^. Further, the functionalizations of cryogel with antimicrobial agents can possess a potential effect against pathogenic bacteria ^52^. Thus, in the current study, the antibacterial activity of a CS-based cryogel was investigated.

**Fig. 8:**
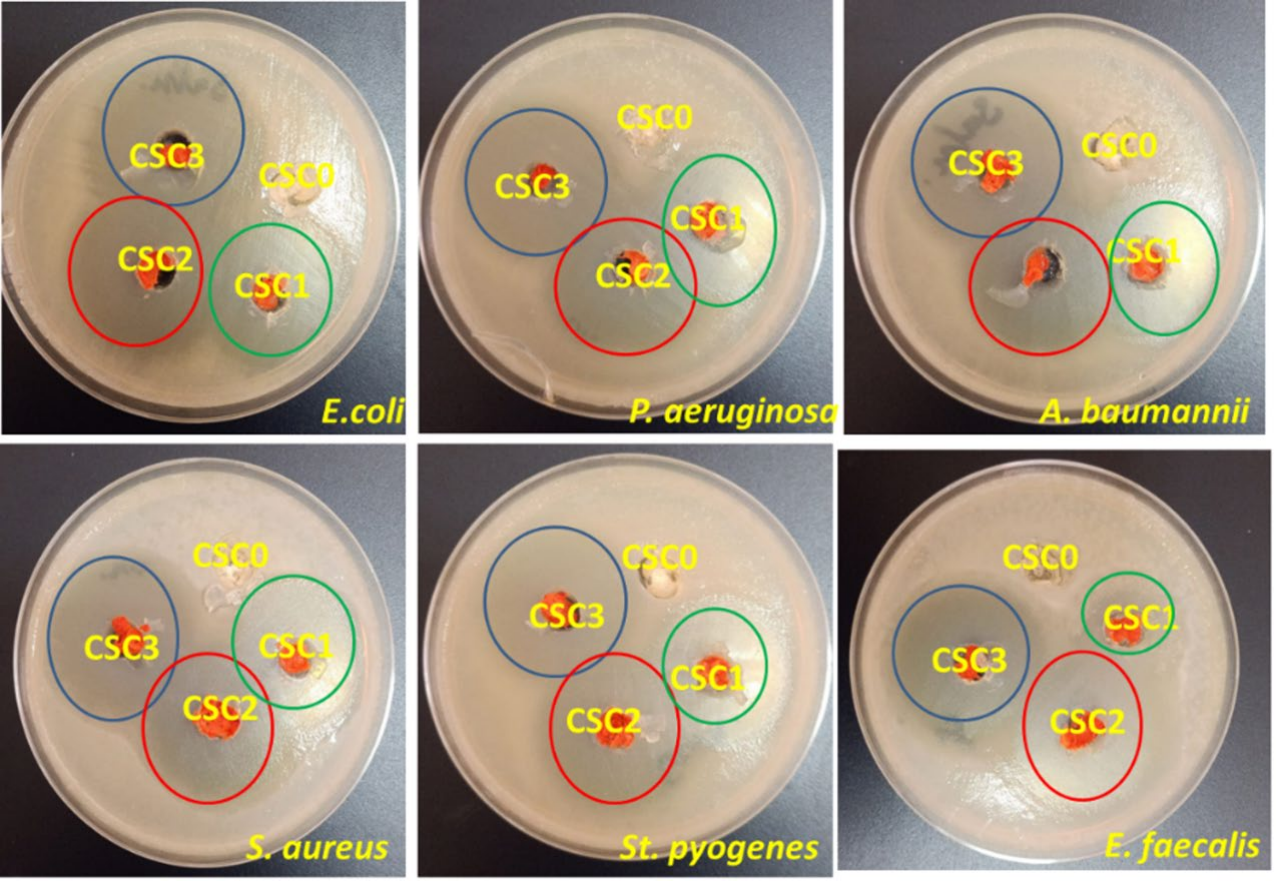
Digital photos of in vitro antibacterial properties using the disc diffusion assay of four cryogels (CSC0, CSC1, CSC2, and CSC3) against some pathogenic bacteria.

The antibacterial efficacy of four CS-based cryogel samples (CSC0, CSC1, CSC2, and CSC3) was evaluated against various bacteria that cause skin infections. This evaluation was conducted using a disk diffusion assay, as seen in Table 2 and Fig. 8. The CSC0 sample (CS without PTZ-chalcone loading) did not show any ZOI against the tested pathogens, indicating the lack of antibacterial activity. Conversely, the ZOI diameters of the CSC1, CSC2, and CSC3 samples, which contained different amounts of PTZ-chalcone (1%, 2%, and 3%, respectively) loaded into CS, showed varying antibacterial activity. The diameters of the ZOI increased with increasing PTZ-chalcone concentration, from CSC1 to CSC2 to CSC3. This suggests that the antibacterial activity of PTZ-chalcone is dependent on its concentration. The CSC2 cryogel exhibited strong antimicrobial activity against various pathogenic bacteria, including *E. coli* (with ZOI diameter of 33.5 ± 0.1 mm), *P. aeruginosa* (with ZOI diameter of 32.4 ± 0.28 mm), *A. baumannii* (with a zone of inhibition diameter of 31.2 ± 0.41 mm), *S. aureus* (with a zone of inhibition diameter of 29.2 ± 0.28 mm), *Str. pyogenes* (with ZOI diameter of 28.7 ± 0.35 mm), and *E. faecalis* (with ZOI diameter of 27.5 ± 0.81 mm). According to the statistical analysis, the differences in ZOI diameters among the tested samples are statistically significant (*p* < 0.05), supporting the concentration-dependent antibacterial activity of PTZ-chalcone-loaded CS cryogels. The results in this article generally indicate that the PTZ-chalcone-loaded CS-based cryogels exhibit promising antibacterial activity.

**Table 2:** Diameters of ZOI (mean ± SD) in mm of the four cryogels against different types of pathogenic bacteria using the disk diffusion assay.

Samples	ZOI diameters in mm					
	E.coli	*P*. aeruginosa	A. baumannii	S. aureus	Str. pyogenes	E.faecalis
**CSC0**	0	0	0	0	0	0
**CSC1**	27 ± 0.5	25 ± 0.35	24.5 ± 0.54	23.3 ± 0.31	22.4 ± 0.52	17.3 ± 0.35
**CSC2**	33.5 ± 0.1	32.4 ± 0.28	31.2 ± 0.41	29.2 ± 0.28	28.7 ± 0.35	27.5 ± 0.81
**CSC3**	32.8 ± 0.3	31.3 ± 0.17	30.7 ± 0.34	28.6 ± 0.54	27.5 ± 0.71	26.3 ± 0.42

The CS cryogels loaded with PTZ-chalcone have potential applications in skin-related therapies, primarily due to their proven antibacterial properties. Initially, it may be inferred that these cryogels may have potential applications in the treatment and management of different skin diseases due to their broad-spectrum antimicrobial action against a variety of skin pathogenic bacteria, such as *E. coli*,* P. aeruginosa*,* A. baumannii*,* S. aureus*,* Str. pyogenes*, and *E. faecalis*. Higher PTZ-chalcone concentrations resulted in more pronounced inhibition zones in the observed concentration-dependent antimicrobial effect. This suggests that the antibacterial effectiveness of these cryogels could be tailored to meet various therapeutic needs. Furthermore, CS is a naturally occurring, biocompatible, and biodegradable polymer that has been extensively studied for wound treatment and drug delivery ^53^. One bioactive ingredient that may significantly improve the therapeutic potential of the CS cryogel matrix for skin-related therapies is PTZ-chalcone ^54^. These PTZ-chalcone loaded CS cryogels, for instance, might be formulated into topical formulations or antimicrobial dressings to treat skin diseases such as ulcers and wounds. Additionally, the cryogel structure could provide an appropriate matrix for the controlled release of the antimicrobial agent, enhancing therapeutic outcomes and reducing the likelihood of resistance development ^55^. Furthermore, due to their antibacterial properties, these cryogels may help treat and prevent skin infections associated with burn injuries, surgical wounds, or damaged skin barriers. Their ability to inhibit the growth of various skin pathogens may help preserve skin health and prevent secondary bacterial infections.

#### Inhibitory impact of cryogel composites

**Fig. 9:**
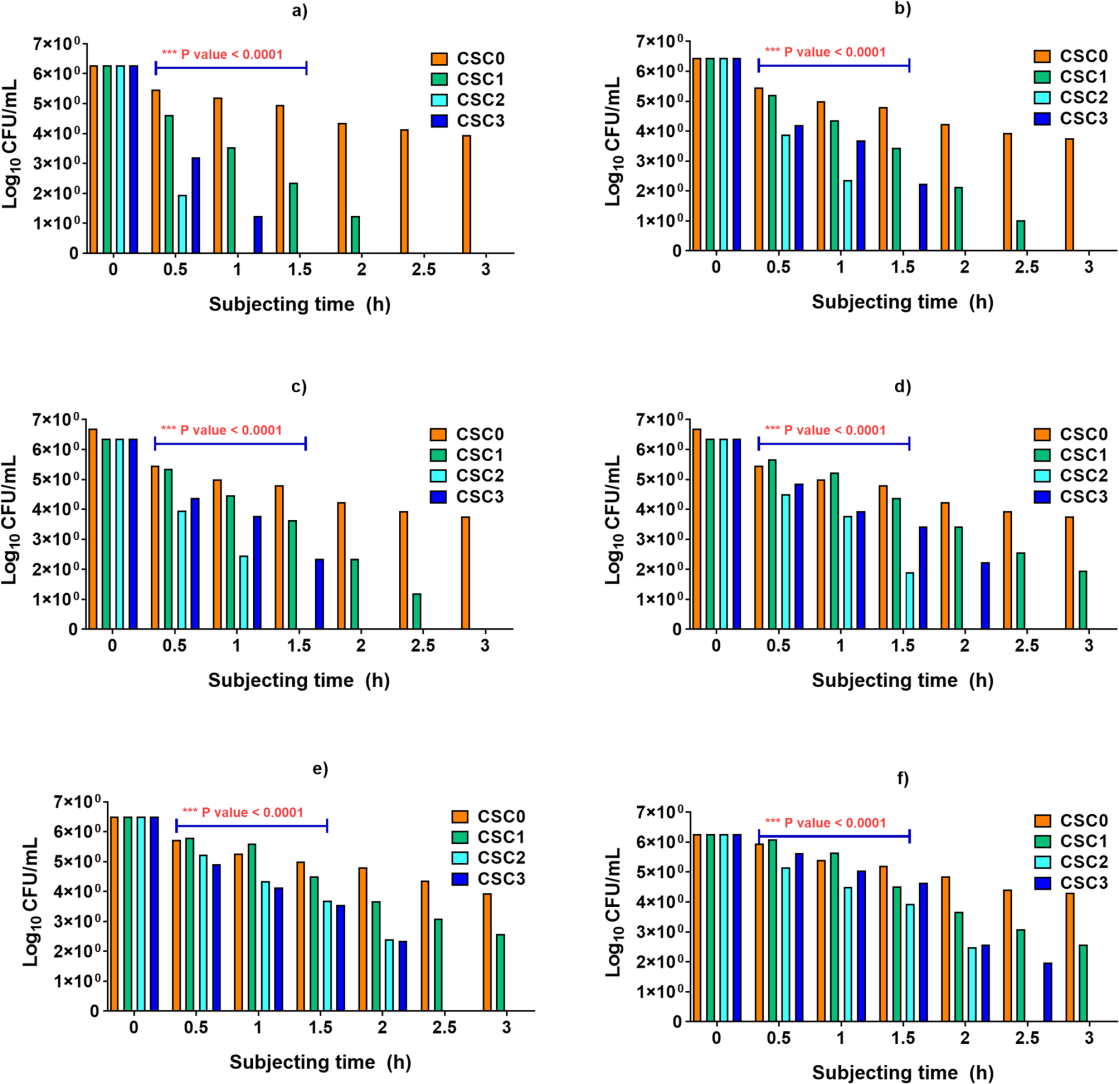
Time-dependent bactericidal activity of prepared cryogel composites (CSC0, CSC1, CSC2, and CSC3*)* against skin pathogenic bacteria, including (a) *E.coli*, (b) *P. aeruginosa*, (c) *A. baumannii*, (d) *S. aureus*, (e) *Str. pyogenes*, and (f) *E. faecalis* at time points (1–6 h).

The time-dependent effects of four cryogel composites (CSC0, CSC1, CSC2, and CSC3) on bacterial survival counts are shown in Fig. 9 (a-f), both before and after loading with the PTZ-chalcone compound. The cryogel composites CSC2 and CSC3 exhibited the most killing efficacy against all tested bacterial strains. These cryogels demonstrated broad-spectrum antibacterial activity, eliminating both Gram-positive and Gram-negative bacteria. The CSC0 sample showed no antibacterial action, this was indicated by the lack of decrease in CFU/mL over time, suggesting that CS was insufficient to combat the examined bacterial strains. In sharp contrast, the CSC2 and CSC3 cryogel composites had the most potent inhibitory effects against certain harmful microorganisms. For each bacterium type, a 6-log drop in CFU was achieved over different times. For *E. coli*, a 1-h exposure was adequate. While *P. aeruginosa* and *A. baumannii* needed 1.5 h, *S. aureus* needed 2 h, while *Str. pyogenes* and *E. faecalis* needed 2.5 h for CSC2 and CSC3. The rapid destruction of bacterial cells by CSC2 and CSC3 suggests that these cryogels damage bacterial cell membranes, leading to cell death. This is probably because the PTZ-chalcone component generates ROS, leading to cell death. The efficacy of PTZ-chalcone-loaded cryogels bacterial cells and inhibiting their proliferation highlights their potential for antibacterial applications. The substantial drop in CFU/mL over time attests to the strong efficacy of these composites in eliminating the bacterial strains, underscoring the improved results achieved by the release and integration of PTZ-chalcone. Furthermore, the effective integration and prolonged release of the PTZ Chalcone molecule, which enhances antibacterial activity, are responsible for the improved efficacy of CSC2 and CSC3. The PTZ-chalcone-loaded cryogels considerably outperformed the unmodified CS cryogel (CSC0), highlighting the importance of cryogel composition in enhancing antimicrobial efficacy. According to these results, a viable method for developing antimicrobial applications, such as wound dressings, is to incorporate active antimicrobial compounds into cryogel composites ^51^.

### Toxicity and biocompatibility performance

The Microtox^®^ test, a standardized technique for assessing the suppression of bioluminescence in the marine bacterium *V. fischeri*, was used to evaluate the toxicity and biocompatibility of the CS-based cryogel composites. For the cryogel samples, the Microtox^®^ EC_50_ (Effective Concentration 50%) values were calculated at exposure durations of 5, 10, and 15 min (Table 3). Lower percentages indicate greater toxicity, while EC_50_ values indicate the toxicity level. The EC_50_ values for the reference toxicity ranges were: 0–19% (highly toxic), 20–39% (very toxic), 40–59% (toxic), 60–79% (mildly toxic), and ≥ 100% (non-toxic and safe). Based on the obtained EC_50_% values, it can be confirmed that all EC_50%_ values exceeded 100, indicating that these cryogel composites were biocompatible and had no toxic potential. Both CSC2 and CSC3 have a favorable biocompatibility profile. A previous study conducted by Pereira et al. ^56^ revealed that the chalcone derivatives are safe and biocompatible. Moreover, the CS hydrogel lacks genotoxic effects ^57^. In particular, its lack of harmful categorization indicates that this formulation is a good option for future research into biomedical applications, including wound-healing bandages. The antibacterial properties of the PTZ-chalcone compound, along with the porous, hydrophilic nature of the CS cryogel, may make this material a useful wound dressing for treating infected or chronic wounds. PTZ-chalcone may help prevent wound infections, and the cryogel matrix may provide a wet, breathing environment to aid healing.

**Table 3:** Measured values of Microtox^®^ EC_50_ % of the prepared cryogel composites.

Cryogels	EC_50_%			References of toxic ranges	
	5 min	10 min	15 min	EC_50_% degree	Toxicity level
CSC0	321	311	301	0–19	Extremely toxic
CSC1	298	284	271	20–39	Very toxic
CSC2	286	272	369	40–59	Toxic
CSC3	284	276	271	60–79	Mild toxic
				≥ 100	Non-toxic and safe

### In silico molecular docking

The binding affinities of the PTZ-chalcone compound with several target proteins, as indicated by ΔG values (kcal/mol), are shown in Table S1 based on the molecular docking studies. The PTZ-chalcone exhibited the highest binding affinity for CDK2, with a ΔG of -9 kcal/mol, indicating a robust interaction. CDK6 exhibited a notable binding affinity, as evidenced by a ΔG value of -8.2 kcal/mol. The molecule exhibited comparable binding affinities for DNA Gyrase B from *P. aeruginosa* and *Str. pyogenes*, with ΔG values of -7.7 and − 7.6 kcal/mol, respectively, suggesting potential antibacterial activity. Among the targets tested, mTOR exhibited the lowest binding affinity, with a ΔG of -6.9 kcal/mol. This suggests that PTZ-chalcone may be less potent against mTOR than against the other targets. The results emphasize the compound’s capacity to inhibit CDK2 and CDK6, which are crucial targets in cancer treatment, and its encouraging antibacterial activity against bacterial DNA Gyrase B ^58^.

### Interaction analysis between PTZ-Chalcone compound and Proteins

**Fig. 10:**
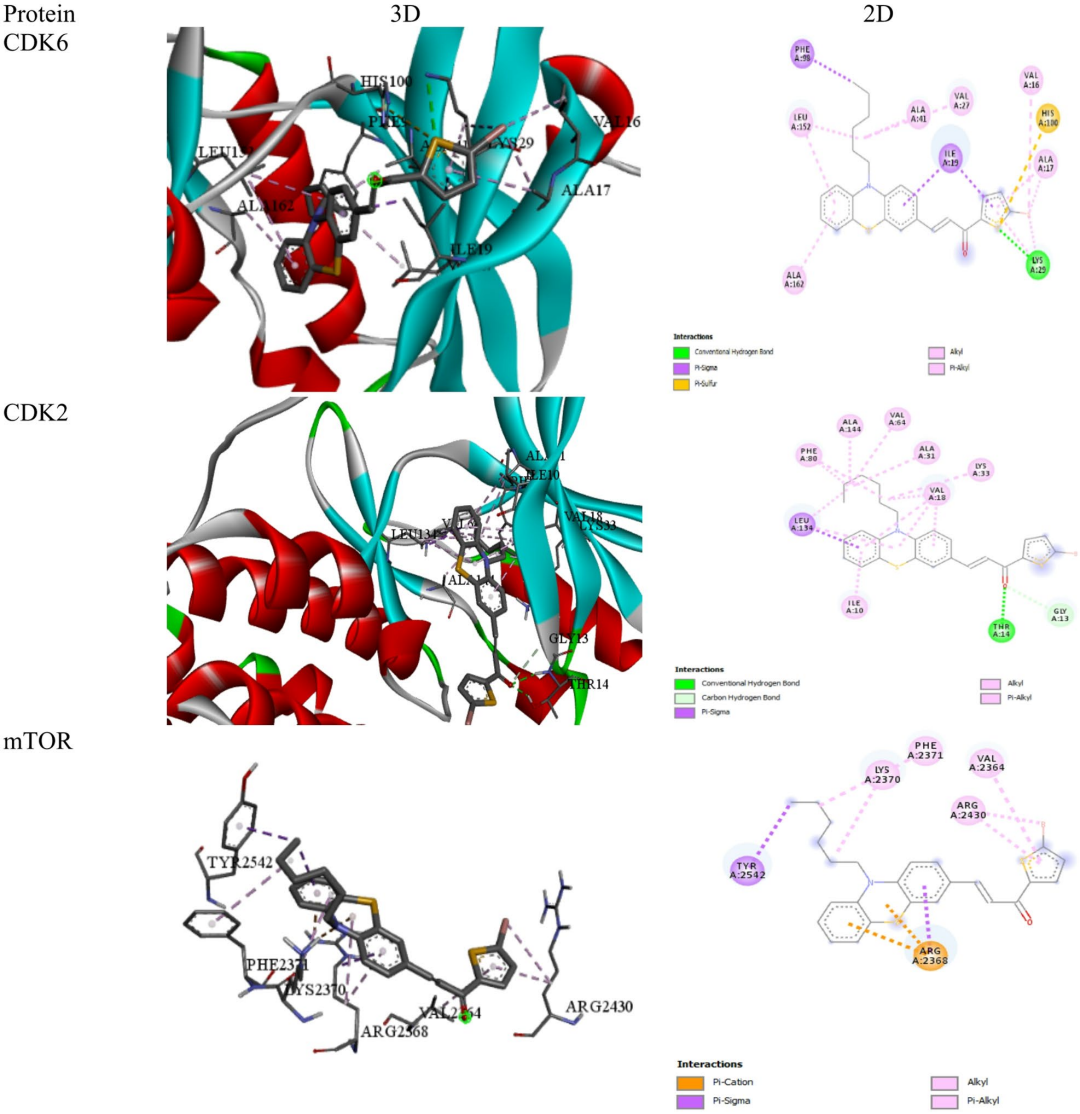

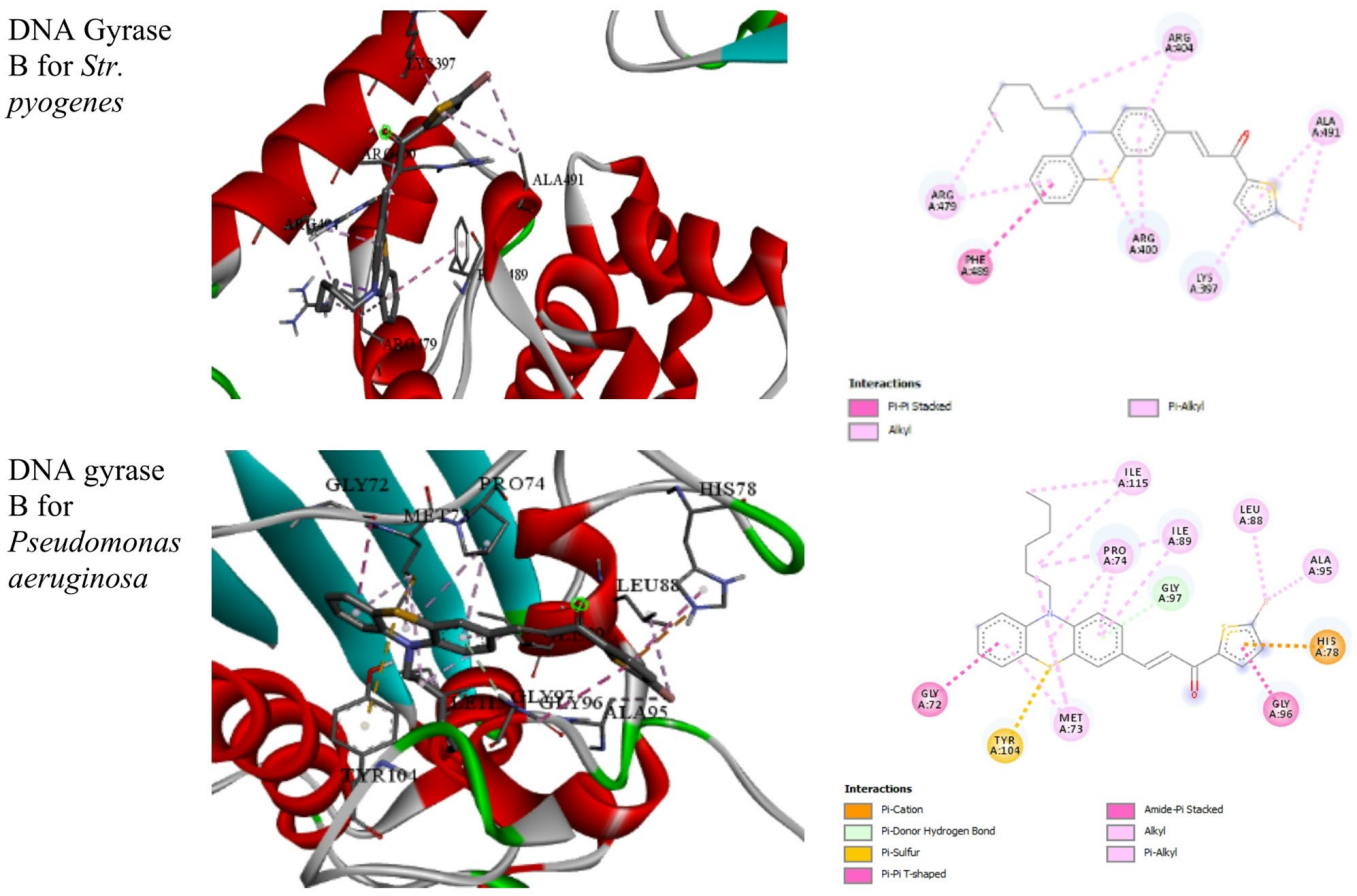
A 3D and 2D interaction between each protein and PTZ.

Figure 10 presents a visual representation of the interactions, both in three dimensions (3D) and two dimensions (2D), between the PTZ-chalcone molecule and several proteins such as CDK6, CDK2, and mTOR. These graphic representations offer comprehensive insights into the specific ways in which molecules bind and the characteristics of their interactions. The 3D structure of CDK6 shows that the PTZ-chalcone fits well within the binding pocket and forms multiple key interactions. The 2D graphic emphasizes specific interactions, such as hydrogen bonds and hydrophobic contacts, involving residues like ARG and ALA, which greatly influence binding affinity. For CDK2, the PTZ-chalcone fits tightly into the binding site, creating a complex network of interactions. The 2D graphic provides detailed information on hydrogen bonds, pi interactions, and hydrophobic contacts, all of which are particularly important to the compound’s strong binding affinity. Notably, these interactions involve residues such as LYS and ASP. The 3D depiction of mTOR shows the precise location of the PTZ-chalcone within the active region, along with the neighboring amino acid residues. The 2D interaction map highlights significant interactions, such as pi-cation and hydrophobic interactions, involving residues like TYR and ARG. Nevertheless, the affinity for mTOR binding is somewhat reduced in relation to CDK2 and CDK6. The interaction patterns highlight the potential of PTZ-chalcone as a protein inhibitor, showing notably high binding affinities for CDK2 and CDK6. The comprehensive interaction maps provide useful insights into the optimization and development of PTZ-chalcone as a medicinal drug targeting these crucial proteins^ 58^.

### The ADMET analysis of the PTZ-chalcone

**Fig. 11:**
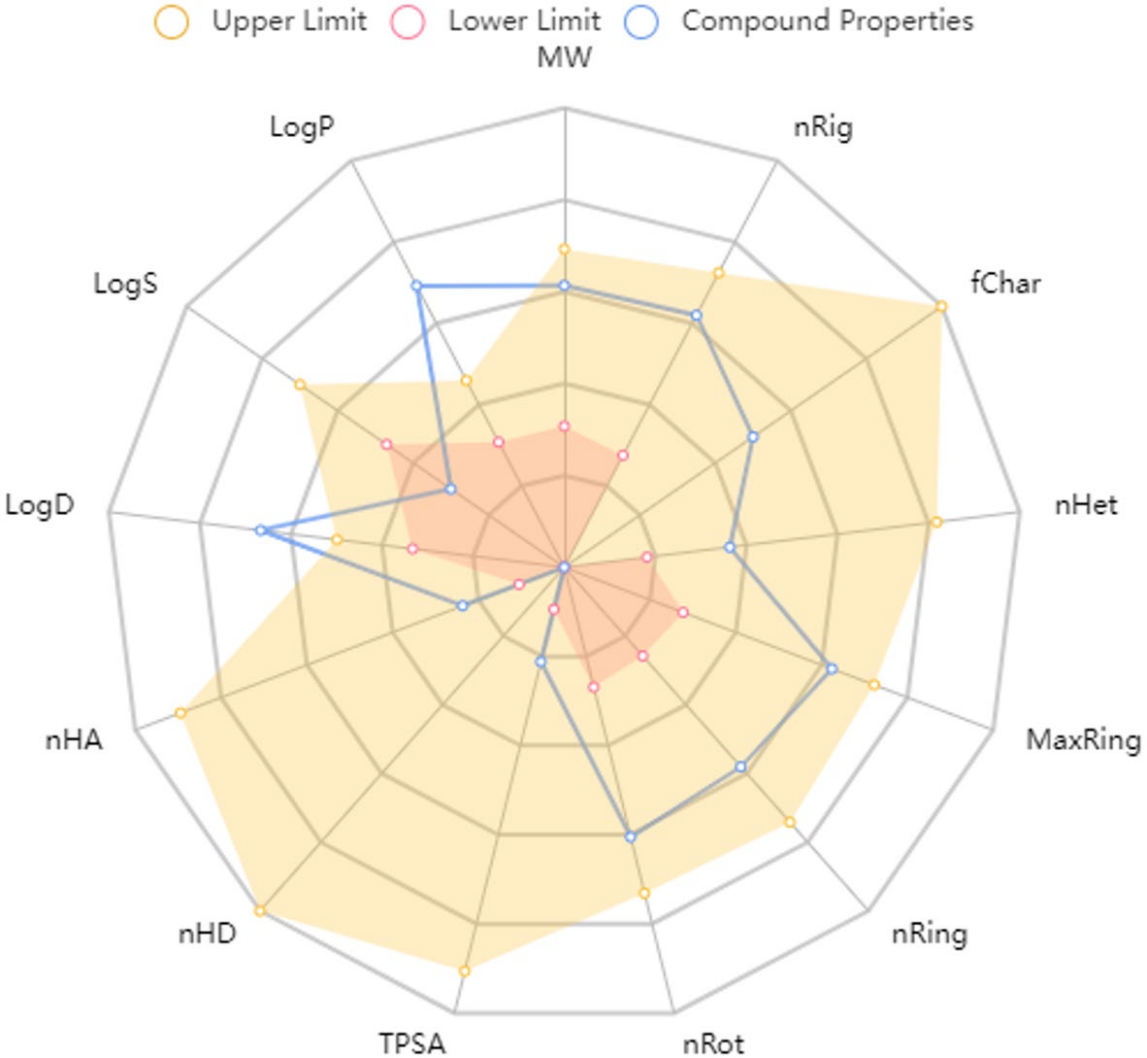
ADMET radar chart of various physicochemical properties of PTZ-chalcone compound, comparing them against upper and lower limits typically associated with drug-like molecules.

This study offers significant findings on the potential of PTZ-chalcone as a medicinal substance for wound dressing purposes, with a specific focus on its pharmacokinetic and toxicological characteristics (Fig. 11 and Table S2). The compound’s molecular weight of 497.05 Da falls within the permitted range for drug-like compounds. The compound’s Log P value of 7.601 suggests a high degree of lipophilicity, which can facilitate its penetration across cell membranes and enable effective delivery to wound areas. The TPSA (Topological Polar Surface Area) value of 20.31 Å² indicates that PTZ-chalcone has a favorable capacity to permeate membranes, making it well-suited for topical applications that require rapid absorption through the skin’s layers. Despite its low water solubility, the compound’s lipophilicity can facilitate its incorporation into lipid-based formulations or delivery methods that enhance skin absorption ^59^.

PTZ-chalcone exhibits a low probability of being a substrate for P-glycoprotein (Pgp-sub: 0.001) (Pgp-inh: 1). This can be advantageous for achieving and sustaining higher therapeutic levels within cells at the site of injury ^60^. The compound has a high plasma protein binding (PPB: 101.81%) and a low percentage unbound (Fu: 1.71%). This suggests that a large amount of the compound can attach to plasma proteins. However, when the compound is applied locally in wound dressings, it is unlikely to enter the bloodstream and have systemic effects. Instead, it will mainly have therapeutic effects in the specific area where it is applied. Regarding metabolism, PTZ-chalcone is expected to be processed primarily via CYP2C9 (0.935) and CYP2D6 (0.904), and to exhibit inhibitory effects on CYP2C19 (0.919) and CYP2C9 (0.931). The metabolic profile indicates that the molecule can be efficiently absorbed through the skin, with little risk of systemic drug interactions or inconsistent exposure. This characteristic is especially advantageous for wound-dressing applications, where targeted and controlled release is essential. Overall, the ADMET properties of PTZ-chalcone indicate its potential efficacy as a constituent of wound dressings. Due to its high lipophilicity, favorable membrane permeability, and capacity to maintain therapeutic concentrations at the site of application, this compound has great potential to improve wound healing and provide antibacterial protection.

Results shown in Fig. 11 indicate that the molecule exhibits drug-like properties but also worrisome attributes, notably elevated lipophilicity (LogP and LogD) and reduced water solubility (LogS), which could affect its bioavailability and pharmacokinetics. The low total polar surface area (TPSA) may suggest favorable membrane permeability.

### Structure-Activity Relationship (SAR) of PTZ-chalcone

The exploration of the SAR provides important insights based on its docking results with CDK6, CDK2, mTOR, and DNA Gyrase B from *Str. pyogenes* and *P. aeruginosa*. The PTZ-chalcone compound has high affinities for CDK6 (-8.2 kcal/mol) and CDK2 (-9 kcal/mol) via hydrogen bonds, hydrophobic contacts, and pi interactions with key amino acid residues, including ARG, LYS, and TYR. The high affinity of PTZ-chalcone may be ascribed to its chemical structure, the α,β unsaturated carbonyl (enone) reacts as an acceptor (Michael acceptor) while the PTZ heterocyclic core, which contains sulfur and N-alkylated heteroatoms, contributes to redox properties and binding ^61,62^. The molecule exhibits modest affinity for DNA Gyrase B (-7.6 to -7.7 kcal/mol) and forms interactions similar to those with CDK targets, although with less stability. The contact between it and mTOR is stabilized by pi-cation and hydrophobic interactions, with a binding energy of -6.9 kcal/mol. The PTZ-chalcone’s high lipophilicity (Log P: 7.601), which is ascribed to the *N*-alkylation of nitrogen heteroatoms in the phenothiazine core, enables it to readily interact with hydrophobic binding sites, thereby improving its binding affinity. However, its lipophilicity may also reduce its solubility and bioavailability. The presence of aromatic rings in the structure facilitates pi-stacking interactions, which are essential for maintaining the stability of complexes with CDK2 and CDK6. Hydrogen bonds further enhance binding strength, especially in CDK targets. The SAR study of PTZ-chalcone demonstrates its potential as a very effective ingredient in wound dressings ^63^. This is due to its strong binding affinities, favorable interactions, and structural characteristics, which contribute to improved wound-healing and antibacterial activity.

## Conclusion

Phenothiazine-based chalcone-loaded chitosan cryogels were successfully synthesized and structurally characterized, confirming the effective incorporation of the PTZ-chalcone derivative within the cryogel matrix. The developed cryogels exhibited a highly porous, interconnected structure and improved thermal stability, supporting their suitability as functional biomaterials. The antibacterial evaluation demonstrated that PTZ-chalcone-loaded cryogels, particularly CSC2 and CSC3, exhibit strong, broad-spectrum bactericidal activity against both Gram-positive and Gram-negative pathogens. Significant zones of inhibition and rapid bacterial reduction confirmed their effective antimicrobial performance. The observed protein leakage further indicated disruption of bacterial membrane integrity as a key mechanism of action. Biocompatibility assessment using the Microtox^®^ assay confirmed that the prepared cryogels are non-toxic and safe under the tested conditions, supporting their potential for biomedical applications. Overall, the results demonstrate that PTZ-chalcone-loaded CS cryogels represent promising antibacterial biomaterials. While the developed cryogels exhibit favorable physicochemical properties (porosity and swelling behavior) and strong antibacterial activity relevant to wound dressing applications.

## Supplementary Information

Below is the link to the electronic supplementary material.

Supplementary Material 1

## Data Availability

The datasets used and/or analyzed during the current study are available from the corresponding author upon reasonable request.
