# Supplementary material for: Phenothiazine dye-loaded chitosan cryogels as multifunctional antibacterial wound dressings
Source: Sci Rep. 2026 Jun 21;16:19196. doi: 10.1038/s41598-026-55319-w (PMC13284323; doi:10.1038/s41598-026-55319-w)
Supplement: Supplementary file 1 — Supplementary Material 1 [file 41598_2026_55319_MOESM1_ESM.docx]

**Supplementary data**

**Phenothiazine Dye-Loaded Chitosan Cryogels as Multifunctional Antibacterial Wound Dressings**

**Reem Ghonaim^1^, Bahaa A.Hemdan^2^, Hesham R. El-Seedi^1,3^, Mohamed M.A. Badr^4^, Tarek S. Aysha^5^, Mervat S. El-Sedik^5^, Samar A. El-Kholy^1^, Mehrez E. El-Naggar^6^.**

^1^Department of Chemistry, Faculty of Science, Menoufia University, Shebin El-Kom 31100107, Egypt

^2^Water Pollution Research Department, Environment and Climate Change Research Institute, National Research Centre, 33 El-Bohouth St., Dokki, Giza 12622, Egypt

^3^International Research Center for Food Nutrition and Safety, Jiangsu University, Zhenjiang, 212013, China

^4^Department of Biochemistry, Faculty of Pharmacy, Menoufia University, Shebin El-Kom 31100107, Egypt;

^5^Dyeing, Printing and Textile Auxiliaries Department, Textile Research and Technology Institute, National Research Centre, 33 EL-Bohouth St., Dokki, Giza, 12622, Egypt.

^6^Pre-Treatment and Finishing of Cellulosic Fabric Department, Textile Research and Technology Institute, National Research Centre, 33 EL-Bohouth St., Dokki, Giza 12622, Egypt.

***Corresponding author:** Tarek Aysha, E.mail:tarekaysha@hotmail.com; Mervat El-Sedik, Mervatelsedik@yahoo.com.


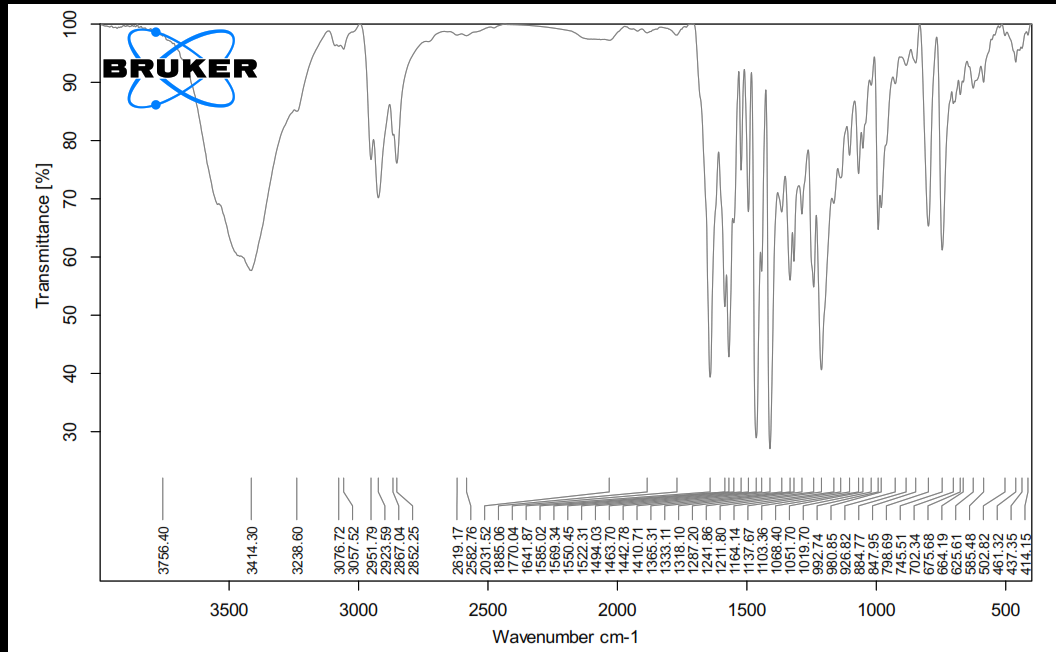


**Figure S1**: FTIR spectrum of the prepared PTZ-chalcone


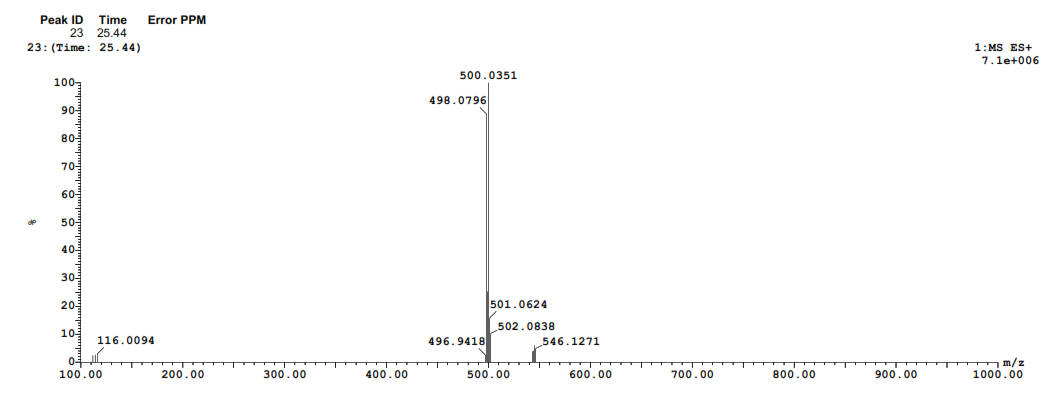


**Figure S2**: ESI positive ion mode mass spectra for the prepared PTZ-chalcone


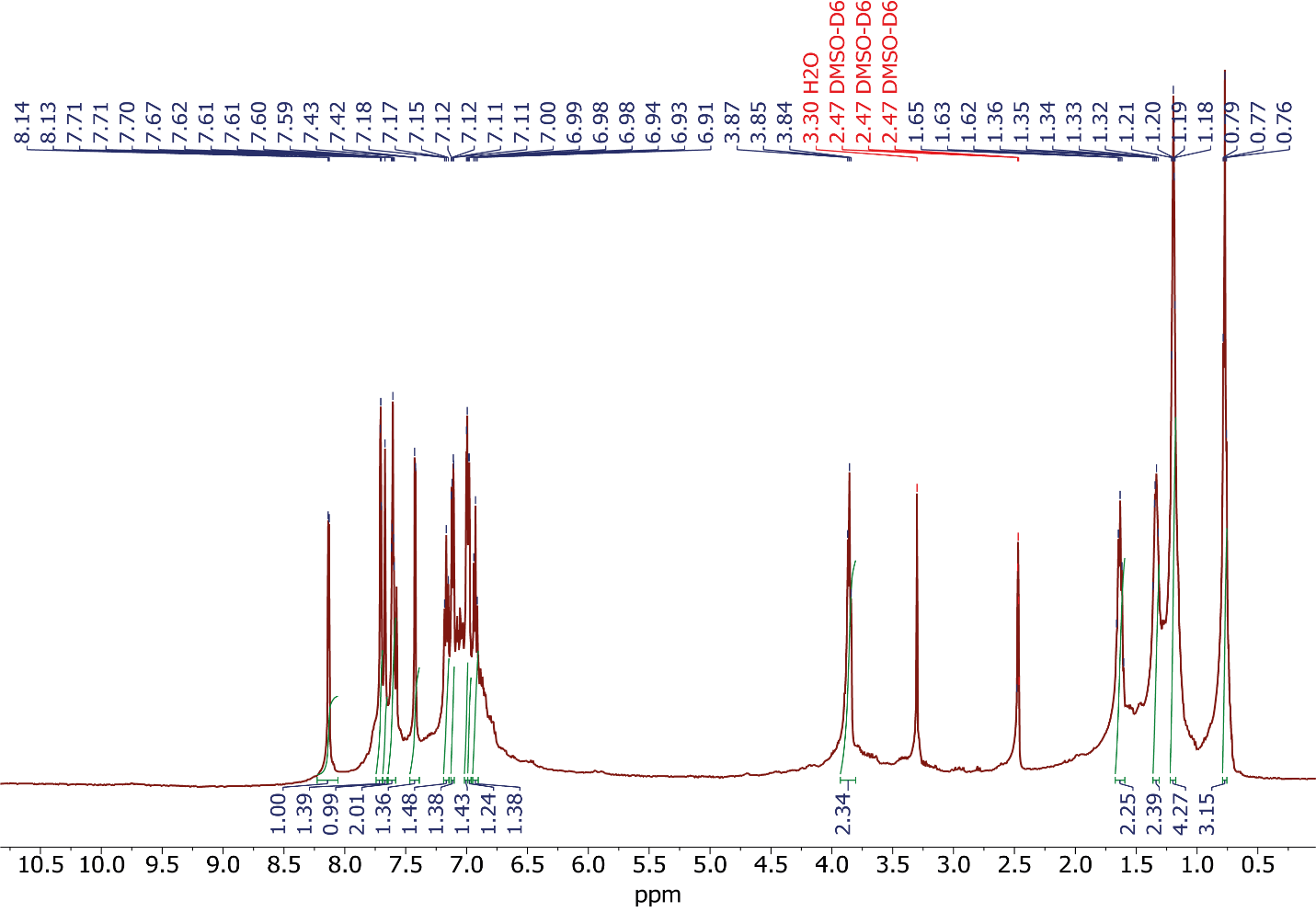


**Figure S3**: the ^1^HNMR spectra of the synthesized PTZ-chalcone

**Figure S4**: the ^13^CNMR spectra of the synthesized PTZ-chalcone

##

**Figure S5:** BET result of neat chitosan cryogel (CSC0) and mofidied one (CSC3).

**Table S1. ∆G (kcal/mol) for each protein**

| **Compound** | **CDK6** | **CDK2** | **mTOR** | **DNA Gyrase B for *Streptococcus pyogenes*** | **DNA gyrase B for *Pseudomonas aeruginosa*** |
| --- | --- | --- | --- | --- | --- |
| PTZ | -8.2 | -9 | -6.9 | -7.6 | -7.7 |

**Table S2**. ADMET properties and Drug likeness for PTZ chalcone compound

| **ADMET** | **Parameter** | **Value** |
| --- | --- | --- |
| Physicochemical Property | LogS | -7.34 |
|  | LogD | 5.017 |
|  | LogP | 7.601 |
| Physicochemical | MW | 497.05 |
|  | Vol | 456.454 |
|  | Dense | 1.089 |
|  | nHA | 2 |
|  | nHD | 0 |
|  | TPSA | 20.31 |
|  | nRot | 8 |
|  | nRing | 4 |
|  | MaxRing | 14 |
|  | nHet | 5 |
|  | fChar | 0 |
|  | nRig | 23 |
|  | Flex | 0.348 |
|  | nStereo | 0 |
| Absorption | Pgp-inh | 1 |
|  | Pgp-sub | 0.001 |
|  | HIA | 0.003 |
|  | F(20%) | 0.011 |
|  | F(30%) | 0.484 |
|  | Caco-2 | -5.034 |
|  | MDCK | 7.97E-06 |
| Distribution | BBB | 0.061 |
|  | PPB | 101.81% |
|  | VDss | 1.65 |
|  | Fu | 1.71% |
| Metabolism | CYP1A2-inh | 0.688 |
|  | CYP1A2-sub | 0.248 |
|  | CYP2C19-inh | 0.919 |
|  | CYP2C19-sub | 0.349 |
|  | CYP2C9-inh | 0.931 |
|  | CYP2C9-sub | 0.935 |
|  | CYP2D6-inh | 0.278 |
|  | CYP2D6-sub | 0.904 |
|  | CYP3A4-inh | 0.632 |
|  | CYP3A4-sub | 0.607 |
| Excretion | CL | 3.721 |
|  | T12 | 0.005 |
| Toxicity | hERG | 0.148 |
|  | H-HT | 0.306 |
|  | DILI | 0.959 |
|  | Ames | 0.443 |
|  | ROA | 0.123 |
|  | FDAMDD | 0.882 |
|  | SkinSen | 0.916 |
|  | Carcinogenicity | 0.463 |
|  | EC | 0.003 |
|  | EI | 0.894 |
|  | Respiratory | 0.472 |
|  | BCF | 1.202 |
|  | IGC50 | 6.023 |
|  | LC50 | 6.831 |
|  | LC50DM | 6.461 |
|  | NR-AR | 0.015 |
|  | NR-AR-LBD | 0.127 |
|  | NR-AhR | 0.839 |
|  | NR-Aromatase | 0.875 |
|  | NR-ER | 0.906 |
|  | NR-ER-LBD | 0.139 |
|  | NR-PPAR-gamma | 0.871 |
|  | SR-ARE | 0.969 |
|  | SR-ATAD5 | 0.903 |
|  | SR-HSE | 0.95 |
|  | SR-MMP | 0.977 |
|  | SR-p53 | 0.934 |
|  | NonGenotoxic_Carcinogenicity | 1 |
|  | LD50_oral | 1 |
|  | Genotoxic_Carcinogenicity_Mutagenicity | 2 |
|  | SureChEMBL | 0 |
|  | NonBiodegradable | 0 |
|  | Skin_Sensitization | 2 |
|  | Acute_Aquatic_Toxicity | 3 |
|  | Toxicophores | 4 |
| Medicinal Chemistry | QED | 0.176 |
|  | Synth | 2.559 |
|  | Fsp3 | 0.24 |
|  | MCE-18 | 42 |
|  | Natural Product-likeness | -0.916 |
|  | Alarm_NMR | 4 |
|  | BMS | 0 |
|  | Chelating | 0 |
|  | PAINS | 0 |
|  | Lipinski | Accepted |
|  | Pfizer | Rejected |
|  | GSK | Rejected |
|  | GoldenTriangle | Rejected |
